# Identification, genotyping, and pathogenicity of *Trichosporon* spp. Isolated from Giant pandas (*Ailuropoda melanoleuca*)

**DOI:** 10.1186/s12866-019-1486-7

**Published:** 2019-05-28

**Authors:** Xiaoping Ma, Yaozhang Jiang, Chengdong Wang, Yu Gu, Sanjie Cao, Xiaobo Huang, Yiping Wen, Qin Zhao, Rui Wu, Xintian Wen, Qigui Yan, Xinfeng Han, Zhicai Zuo, Junliang Deng, Zhihua Ren, Shumin Yu, Liuhong Shen, Zhijun Zhong, Guangneng Peng, Haifeng Liu, Ziyao Zhou

**Affiliations:** 1 Key Laboratory of Animal Disease and Human Health of Sichuan Province, College of Veterinary Medicine, Sichuan Agricultural University, Chengdu, 611130 China; 2 China Conservation and Research Center for the Giant Panda, Ya’an, 625000 Sichuan China; 3 College of Life Sciences, Sichuan Agricultural University, Chengdu, 611130 China

**Keywords:** *Trichosporon*, ITS, D1/D2;IGS1, Identification, Morphology, Pathogenicity

## Abstract

**Background:**

*Trichosporon* is the dominant genus of epidermal fungi in giant pandas (*Ailuropoda melanoleuca*) and causes local and deep infections. To provide the information needed for the diagnosis and treatment of trichosporosis in giant pandas, the sequence of ITS, D1/D2, and IGS1 loci in 29 isolates of *Trichosporon* spp. which were isolated from the body surface of giant pandas were combination to investigate interspecies identification and genotype. Morphological development was examined via slide culture. Additionally, mice were infected by skin inunction, intraperitoneal injection, and subcutaneous injection for evaluation of pathogenicity.

**Results:**

The twenty-nine isolates of *Trichosporon* spp. were identified as 11 species, and *Trichosporon jirovecii* and *T. asteroides* were the commonest species. Four strains of *T. laibachii* and one strain of *T. moniliiforme* were found to be of novel genotypes, and *T. jirovecii* was identified to be genotype 1. *T. asteroides* had the same genotype which involved in disseminated trichosporosis. The morphological development processes of the *Trichosporon* spp. were clearly different, especially in the processes of single-spore development. Pathogenicity studies showed that 7 species damaged the liver and skin in mice, and their pathogenicity was stronger than other 4 species. *T. asteroides* had the strongest pathogenicity and might provoke invasive infection. The pathological characteristics of liver and skin infections caused by different *Trichosporon* spp. were similar.

**Conclusions:**

Multiple species of *Trichosporon* were identified on the skin surface of giant panda, which varied in morphological development and pathogenicity. Combination of ITS, D1/D2, and IGS1 loci analysis, and morphological development process can effectively identify the genotype of *Trichosporon* spp.

**Electronic supplementary material:**

The online version of this article (10.1186/s12866-019-1486-7) contains supplementary material, which is available to authorized users.

## Background

The giant panda (*Ailuropoda melanoleuca*) is one of the rarest endangered animals[1, 2]. Dermatomycosis has become the second major disease of giant pandas, which seriously affect the survival of giant pandas [3]. Our previous studies have shown that *Trichosporon* spp. is the dominant genera of the body surface [4] and genitals of giant pandas [2]. These species may lead to trichosporidiosis in humans and animals.

*Trichosporon* is a genus of fungi that belongs to the order *Tremellales* in the class *Tremellomycetes* (division *Basidiomycota*) and is widely distributed in nature [5]. *Trichosporon* spp. can cause superficial fungal infections such as *tinea pedis*, onychomycosis, and dermoid infections [6]. With the increasing prevalence of immunocompromised patients, the incidence of invasive fungal diseases has increased, and *Trichosporon* has become the second commonest genus of yeast in deep fungal infections in patients with hematologic malignancies, granulocytic deficiency, and bone marrow transplants [7]. Meanwhile, *Trichosporon* spp. infections of animals have increased, such as disseminated trichosporosis in cats [8], canine meningitis [9], and tortoise cutaneous infection [10]. Owing to the difficulties in classification and identification of *Trichosporon* spp., studies on them have substantially lagged behind other species in many areas such as clinical characteristics, antifungal susceptibilities, and the selection of therapeutic drugs [11]. To accurately identify *Trichosporon* spp., a number of molecular methods have been developed, of which DNA sequencing of the internal transcribed spacer (ITS) region, the D1/D2 domain of the 26S subunit of the rRNA gene region, and the intergenic spacer 1 (IGS1) region are the most frequently used. The IGS1 gene region is particularly useful in phylogenetic studies and the description of intraspecies variation [12, 13]. Ribeiro et al. identified 21 clinical isolates as belonging to six species on the basis of the ITS and IGS1 regions [14]. In 2009, Chagas Neto et al. identified 22 isolates from human blood by analyzing the IGS1 region [15]. In 2011, Guo identified 29 clinical isolates of *Trichosporon* by analyzing 3 loci, and eight *Trichosporon* spp. were found, of which *Trichosporon asahii* was the commonest [13].

Morphology study also used to identify *Trichosporon* spp., but the result was not revealing. Li performed slide culture of six clinically common *Trichosporon* spp. and found no significant differences in colony morphology [16].

*Trichosporon* spp. are the dominant fungal species on giant panda skin and genitals [4]. Meanwhile, there are no reports on systemic identification of *Trichosporon* spp. isolated from animals. So identification *Trichosporon* spp. at the species level is important for preventing and treating dermatomycoses in giant pandas.

We collected 29 isolates of *Trichosporon* spp. from the skin of giant pandas breeding at the China Conservation and Research Center for Giant Pandas, Ya’an. Owing to the shortage of sequence data for the IGS1 region in individual species, we had to analyze the ITS region, D1/D2 domain, and IGS1 region of the isolates to obtain accurate classification information. Afterward, the morphological development process was observed by the slide culture method. Mice were artificially infected, and their livers and skin were taken for pathological analysis.

## Methods

### Sampling procedure

Samples were collected from clinically healthy giant pandas (22 females and 22 males) at the China Conservation and Research Center for Giant Pandas (Ya’an, China) in 2015–2016. Pandas that had been treated with antifungal drugs during the previous 6 months or with a recent history of disease were excluded from this study. The pandas lived in a semi-captive semi-enclosed breeding environment, were fed a diet of about 10% steamed cornbread and fruits and 90% bamboo shoots, and were allowed to drink water *ad libitum *[2].

All personnel involved in sampling wore sterile protective clothing, hats, masks, and latex gloves. Use sterilized shears to remove most of the hair when a panda ate fruits, 70% alcohol was used to sterilize the surface of the upper back of forearm wrist (5.0 cm × 5.0 cm, approximately), use the edge of the sterilized scalpel to scrape the surface and then a suitable amount of dander were collected. All samples were quickly placed in sterilized plastic sample bags, transported to the laboratory on ice within 2 h, and then immediately processed in a BSL-2 safety cabinet. No repeat sampling was performed on the same panda, and all 44 samples were processed for isolating *Trichosporon* spp.[2].

### Fungal culture

Samples were streak-inoculated under aerobic conditions onto Sabouraud dextrose agar (SDA) (MOLTOX, Inc., Boone, NC) containing 4% (m/v) glucose, 1% (m/v) peptone, and 1.5% (m/v) agar. When samples were first inoculated, media were supplemented with chloramphenicol (0.005%, m/v). The chloramphenicol are not added to the medium in subsequent culture.

Fungal culture was carried out in a BSL-2 safety cabinet in a bioclean room. Sterilized sealing film was used to cover each plate. Each sample was plated onto three culture plates with three control plates. All culture dishes were inoculated and stored at 25 °C for 7–30 days before being considered negative [2].

### Molecular identification

Fungal DNA was extracted from pure culture as described previously [17]. Amplification of the ITS region, D1/D2 domain, and IGS1 region was performed as described with the primer pairs ITS1/ITS4 (ITS1: 5′-TCCGTAGGTGAACCTGCGG-3′; ITS4: 5′-TCCTCCGCTTATTGATATGC-3′), F63/R635 (F63: 5′-GCATATCAATAAGCGGAGCAAAAG-3′; R635: 5′-GGTCCGTGTTTCAAGACG-3′), and 26SF/5SR (26SF: 5′-ATCCTTTGCAGACGACTTGA-3′; 5SR: 5′-AGCTTGACTTCGCAGATCGG-3′), respectively [12, 18]. PCR amplification was performed in a 50 μl reaction mixture containing 19 μl 2 × Taq Master Mix (Tsingke Biotech Co., Ltd., Chengdu, China), 2 μl primers, 25 μl double-distilled water, and 2 μl fungal genomic DNA. The thermocycling conditions were as follows: 5 min at 98 °C (initial denaturation), 35 cycles of 10 s at 98 °C, 10 s at 58 °C, and extension at 72 °C for 10 s, and final extension for 4 min at 72 °C. A total of 8 μl of the amplified PCR products were visualized on 2% agarose gel after staining with GreenView (Solarbio, Beijing). The PCR products were then sequenced by Tsingke Biotech Co., Ltd. (Chengdu, China).

All the chromatograms of DNA sequences were examined to ensure high-quality sequences. For species identification, the sequences of the ITS region, D1/D2 domain, and IGS1 region were queried against the NCBI database (https://www.ncbi.nlm.nih.gov/genbank). The sequence of each locus and concatenated sequences were then aligned using the NCBI BLAST and formed the consensus sequences for all 29 isolates. Phylogenetic trees were computed with MEGA version 6 (Molecular Evolutionary Genetic Analysis software version 6.0.2; http://www.megasoftware.net) using the neighbor-joining method, in which all positions containing gaps and missing data were eliminated from the dataset. The ITS region plus the D1/D2 domain and IGS1 region were used to produce two separate phylogenetic trees. All sequences of the three genes from the 29 isolates were deposited in the GenBank database (https://www.ncbi.nlm.nih.gov/genbank/) and were assigned ID numbers (Table 1).

**Table 1:** *Nucleotide sequence accession numbers*

Strain	Molecular Identification	IGS1 blast	ITS GenBank Accession Number	D1/D2 GenBank Accession Number	IGS1 GenBank Accession Number
JYZ3252	*T. laibachii*	*T. laibachii*	KX302021	MG708435	MG708464
JYZ921	*T. laibachii*	*T. laibachii*	KX034345	MG708441	MG708470
JYZ321	*T. laibachii*	*T. laibachii*	KX302022	MG708433	MG708462
JYZ912	*T. laibachii*	*T. laibachii*	KX034344	MG708439	MG708468
JYZ1291	*T. gracile*	*T. gracile*	KX302008	MG708454	MG708483
JYZ1253	*T. brassicae*	*T. brassicae*	KX302047	MG708450	MG708479
JYZ983	*T. domesticum*	*T. domesticum*	KX034390	MG708444	MG708473
JYZ1221	*T. guehoae*	*Trichosporon* sp.	KX302031	MG708445	MG708474
JYZ1224	*T. guehoae*	*Trichosporon* sp.	KX302081	MG708447	MG708476
JYZ915	*T. guehoae*	*Trichosporon* sp.	KX034350	MG708440	MG708469
JYZ1251	*T. asteroides*	*T. asteroides*	KX302012	MG708448	MG708477
JYZ1281	*T. asteroides*	*T. asteroides*	KX302074	MG708453	MG708482
JYZ371	*T. asteroides*	*T. asteroides*	KX302060	MG708437	MG708466
JYZ1255	*T. asteroides*	*T. asteroides*	KX302051	MG708451	MG708480
JYZ951	*T. asteroides*	*T. asteroides*	KX034347	MG708443	MG708472
JYZA10	*T. jirovecii*	*T. jirovecii*	MG857660	MG708460	MG708489
JYZA5	*T. jirovecii*	*T. jirovecii*	MG857657	MG708457	MG708486
JYZA7	*T. jirovecii*	*T. jirovecii*	MG857658	MG708458	MG708487
JYZA9	*T. jirovecii*	*T. jirovecii*	MG857659	MG708459	MG708488
JYZA12	*T. jirovecii*	*T. jirovecii*	MG857661	MG708461	MG708490
JYZ030202	*T. cutaneum*	*T. cutaneum*	MG857656	MG708456	MG708485
JYZ1223	*T. shinodae*	no result	KX302045	MG708446	MG708475
JYZ1261	*T. middelhovenii*	no result	KX302046	MG708452	MG708481
JYZ1252	*T. middelhovenii*	no result	KX302043	MG708449	MG708478
JYZ12922	*T. middelhovenii*	no result	KX302044	MG708455	MG708484
JYZ932	*T. moniliiforme*	*T. moniliiforme*	KX034371	MG708442	MG708471
JYZ331	*T. moniliiforme*	*T. moniliiforme*	KX302017	MG708436	MG708465
JYZ372	*T. moniliiforme*	*T. moniliiforme*	KX302067	MG708438	MG708467
JYZ323	*T. moniliiforme*	*T. moniliiforme*	KX302018	MG708434	MG708463

Note: Twenty-nine isolates of *Trichosporon* spp. were identified as belonging to 11 species, namely, *T. laibachii* (4 isolates), *T. gracile* (1 isolate), *T. brassicae* (1 isolate), *T. domesticum* (1 isolate), *T. guehoae* (3 isolates), *T. asteroides* (5 isolates), *T. jirovecii* (5 isolates), *T. cutaneum* (1 isolate), *T. shinodae* (1 isolate),

*T. middelhovenii* (3 isolates), and *T. moniliiforme* (4 isolates). *T. jirovecii* and *T. asteroides* were the commonest species, each of which accounted for 17% (5/29) of the isolates

### Morphological development process

Microscopic observations of the 29 isolates were made after slide culture on SDA [5]. A 0.5 ml sample of melted medium was injected into a closed glass Petri dish, which comprised a slide glass, a cover glass, and a copper ring with a hole in the wall, and was inoculated via the hole [19, 20]. All isolates were incubated at 25 °C and were observed after 24, 48, 72 and 96 h. The cover glass was stained with 5 ml Lactophenol cotton blue (Hopebio, Qingdao, China) and was observed with a microscope (BX51, Olympus).

### Pathogenicity experiment

#### Animal experiment

In total of 216 sex-matched SPF Kunming mice (Dashuo Science and Technology Co., Ltd., China), which belonged to 11 experiment groups and one control group, with ages of 6–8 weeks were used. Each *Trichosporon* sp. was inoculated by skin inunction (We abraded skin with emery paper until a slight bleeding and cut the hair (2 cm × 2 cm) on the back of mouse after anesthesia by diethyl ether), subcutaneous injection, and intraperitoneal injection into immunosuppressed and non-immunosuppressed mice. In total, six groups were used, each of which comprised three mice. Groups A (intraperitoneal injection), B (subcutaneous injection), and C (skin inunction) were immunosuppressed (Mice were given intraperitoneal injection with 50 mg/kg cyclophosphamide at intervals of 48 h, three times in total, and each mouse was given 15 mg of penicillin sodium under the skin.); groups D (intraperitoneal injection), E (subcutaneous injection), and F (skin inunction) were non-immunosuppressed.

#### Preparation of fungal suspension and inoculation

Before being inoculated, mice in the immunosuppressed groups were intraperitoneally injected with *Trichosporon* spp. that had been cultured in SDA at 25 °C for 5 d. The mycelium and spores were scraped, washed with physiological saline, and mixed well. A hemocytometer was used to adjust the concentration of the spore suspension to 1 × 10^7^ CFU/ml. Except for control group, each mouse was inoculated with 0.1 ml fungal suspension. Mice in the control group received 0.1 ml physiological saline instead. The backs of the mice treated by skin inunction were shaved, sterilized with 75% (w/w) alcohol, and lightly abraded with a 25G needle, and then 0.1 ml fungal suspension was gently rubbed onto the skin with a sterile injector.

#### Tissue sample processing

The ingestion and clinical symptoms of the mice were observed daily. The mice were anesthetized and euthanized with 5 ml diethyl ether (Chengdu Kelong Chemical Reagent Factory, Chengdu, China) using anesthetic gas box (AC-100, Yuyan Instruments, Shanghai), decapitated, and dissected to observe lesions on the seventh day after infection. The livers of mice in groups A and D and skin lesions from mice in the other groups were taken for fungal culture and pathological evaluation. The livers and skin were placed in 100 ml 4% formalin (w/w) (Chengdu Kelong Chemical Reagent Factory; Chengdu, China) for histopathological study via staining with hematoxylin/eosin (HE) and periodic acid/Schiff stain (PAS)[9].

## Results

### Molecular identification and genotyping

**Fig. 1:**
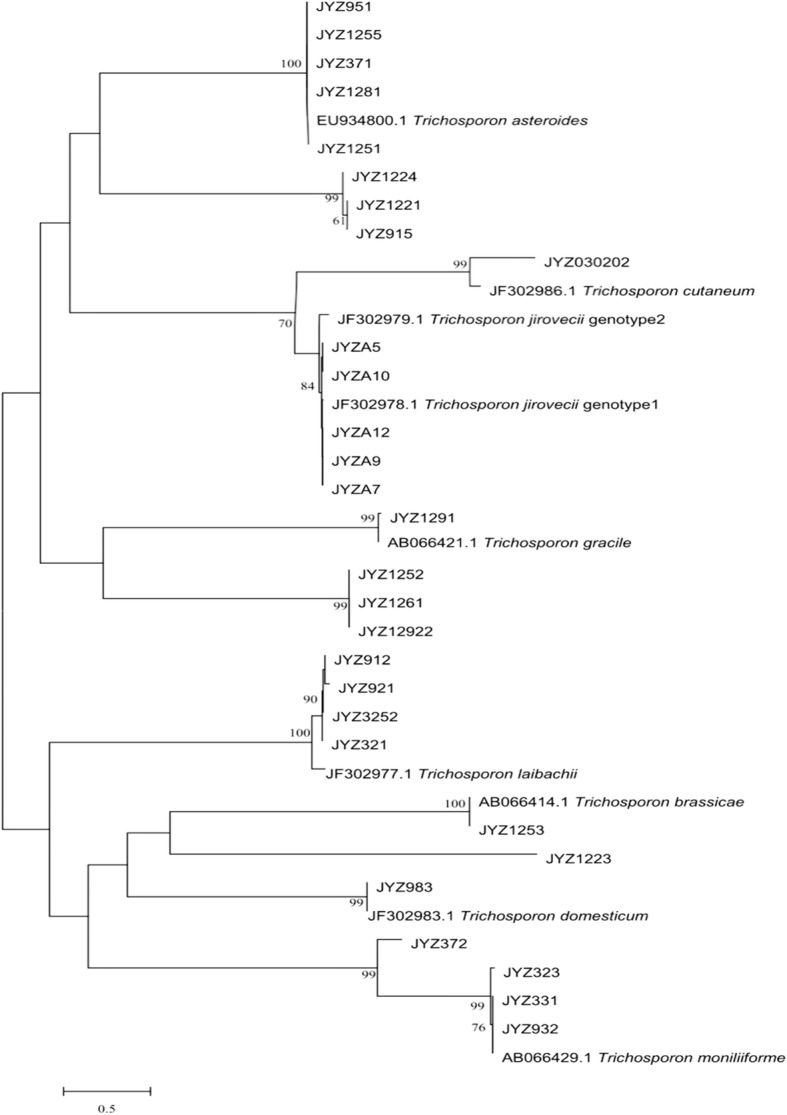
Phylogenetic tree based on IGS1 sequences

**Fig. 2:**
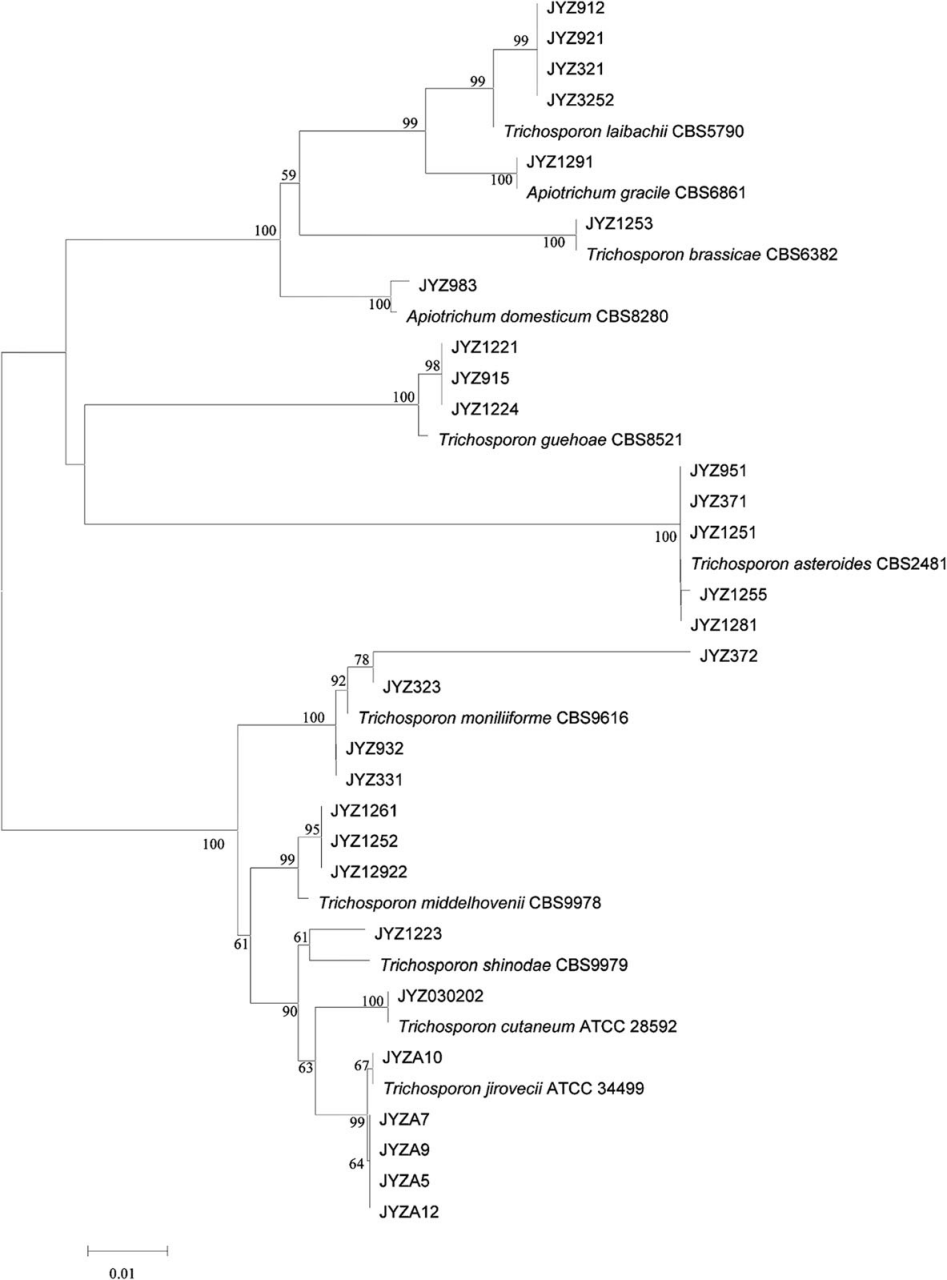
Phylogenetic tree based on ITS sequences plus D1/D2 sequences

The interspecies identification of 22 isolates of *Trichosporon* spp. was performed from the data in Fig. 1. Seven strains of *Trichosporon* (JYZ1221, JYZ1224, JYZ915, JYZ1223, JYZ1261, JYZ1252, and JYZ12922) could not be identified because of the lack of sequence information for IGS1 in the GenBank database. However, it was determined that these seven *Trichosporon* strains belonged to three species. The interspecies identification of all 29 isolates of *Trichosporon* spp. was performed using the data in Fig. 2. The structures of the two phylogenetic trees were basically identical, and the method could be used to determine the accuracy of the identification of the 29 isolates. The 29 isolates of *Trichosporon* spp. were identified as belonging to 11 species, namely, *T. laibachii* (4 strains), *T. gracile* (1), *T. brassicae* (1), *T. domesticum* (1), *T. guehoae* (3), *T. asteroides* (5), *T. jirovecii* (5), *T. cutaneum* (1), *T. shinodae* (1), *T. middelhovenii* (3), and *T. moniliiforme* (4). *T. jirovecii* and *T. asteroides* were the commonest species (17%, 5/29). Moreover, *T. middelhovenii* and *T. shinodae* were isolated from the surfaces of animals for the first time.

In this study, preliminary genotyping was performed on *Trichosporon* spp. *T. asteroides* (JYZ1251, JYZ1281, JYZ371, JYZ1255, and JYZ951) had the same genotype as its reference strain, which was isolated from the blood of immunocompromised patients [15], whereas *T. laibachii* (JYZ3252, JYZ921, JYZ321, and JYZ912) had different genotypes. The reference strain was isolated from humans. The five isolates of *T. jirovecii* were identified as having genotype 1[13]. *T. brassicae* (JYZ1253) had the same genotype as the reference strain, which was isolated from cabbage [12]. *T. gracile* (JYZ1291) had the same genotype as the reference strain, which was isolated from spoiled milk [12]. *T. domesticum* (JYZ983) and the reference strain had the same genotype; the reference strain was isolated from human sputum [12]. Three strains of *T. moniliiforme* (JYZ932, JYZ331, and JYZ323) had the same genotype as the reference strain, which was isolated from curdling milk [12], but the strain JYZ372 did not have this genotype. It was difficult to determine whether *T. cutaneum* had the same genotype as the reference strain. According to the phylogenetic tree, the genetic relationship was distant and it may not have had the same genotype.

### Morphological development process

All 29 isolates were identified as *Trichosporon* spp. after molecular identification. The morphological development processes of the *Trichosporon* spp. were clearly different, and the difference was significant for the processes of single-spore development. Spores of *T. moniliiforme* and *T. shinodae* tended to be self-replicating at the beginning of their development and reproduced mainly by budding. Spores of the other species tended to become mycelia and reproduced mainly by the differentiation of mycelia to form spores or produce arthrospores. Morphological development could be used as an important basis for the identification of *Trichosporon* spp. For example, *T. moniliiforme* produced a large number of spores, and the spores were transformed into an oval shape after development was completed. The growth of *T. shinodae* was the slowest among the *Trichosporon* spp.; the shape of mycelia was specific and served as a basis for identification. *T. laibachii* produced a large number of arthrospores and had the unique feature that the mycelia were folded together. The main method of growth of *T. guehoae* comprised the reproduction of arthrospores by budding, and it had the specific feature that new mycelia grew from gaps in segmented mycelia. The morphological appearances of *T. brassicae*, *T. domesticum* and *T. gracile* were analogous in some ways. All these *Trichosporon* spp. tended to reproduce via the differentiation of mycelia into spores, and few arthrospores were seen during the process of development. The shapes of spores that differentiated from mycelia were different: those of *T. gracile* were quadrilateral, those of *T. domesticum* were round, and those of *T. brassicae* were disciform. The arthrospores of *T. middelhovenii* were fusiform and were always located in a bifurcation of the mycelium. This characteristic was distinct from other *Trichosporon* spp. *T. asteroides* had the feature that the spores overlapped each other, and the mycelia were thin and short. The mycelia could differentiate into spores, and the pigmentation of the spores was uneven, as shown by dyeing with cotton blue. The morphologies of *T. jirovecii* and *T. cutaneum* were similar, but the mycelia of *T. cutaneum* were more curved and tended to differentiate into spores, in contrast to *T. jirovecii*. *Trichosporon* spp. have individual morphological characteristics and hence could be distinguished by means of a comparison of their morphological development processes.

**Fig. 3:**
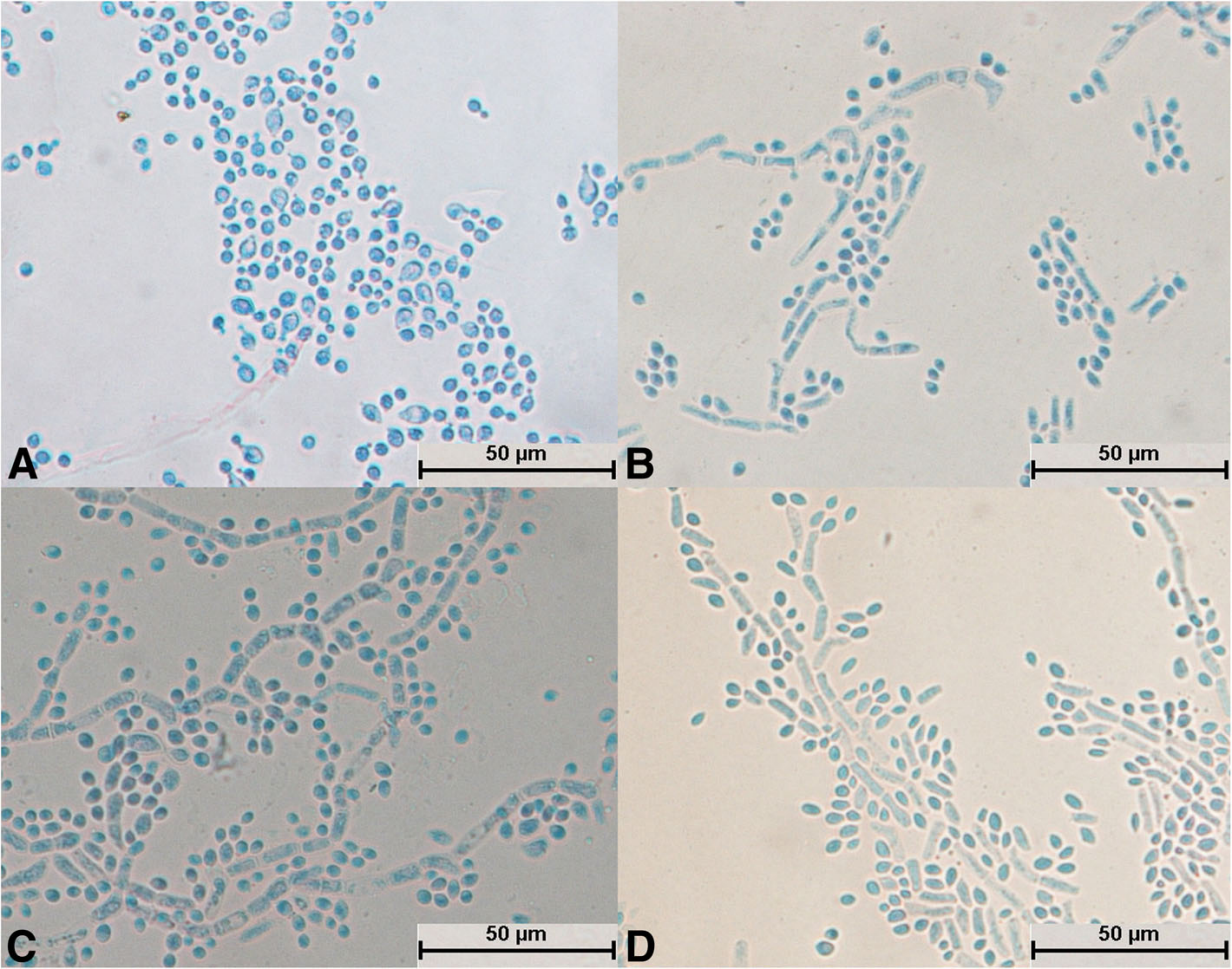
Morphological development process of *Trichosporon moniliiforme* from day 1 to day 4. **a** Most spores divided and a small number of spores expanded; **b**: Scattered hyphae appeared and began to produce conidia on day 2; **c**: Basic hyphae formed; **d**: Spores increased in number and their shapes were transformed from circular to elliptical

After culture for 24 h, spores of *T. moniliiforme* divided independently (Fig. 3a), no hyphae differentiated into spores (Fig. 3b, c, and d), and the mycelium and spores were evenly stained (Fig. 3b, c, and d). The mycelium produced a large number of arthrospores (Fig. 3c), and the shape of the spores changed from circular (Fig. 3c) to elliptical (Fig. 3d).

**Fig. 4:**
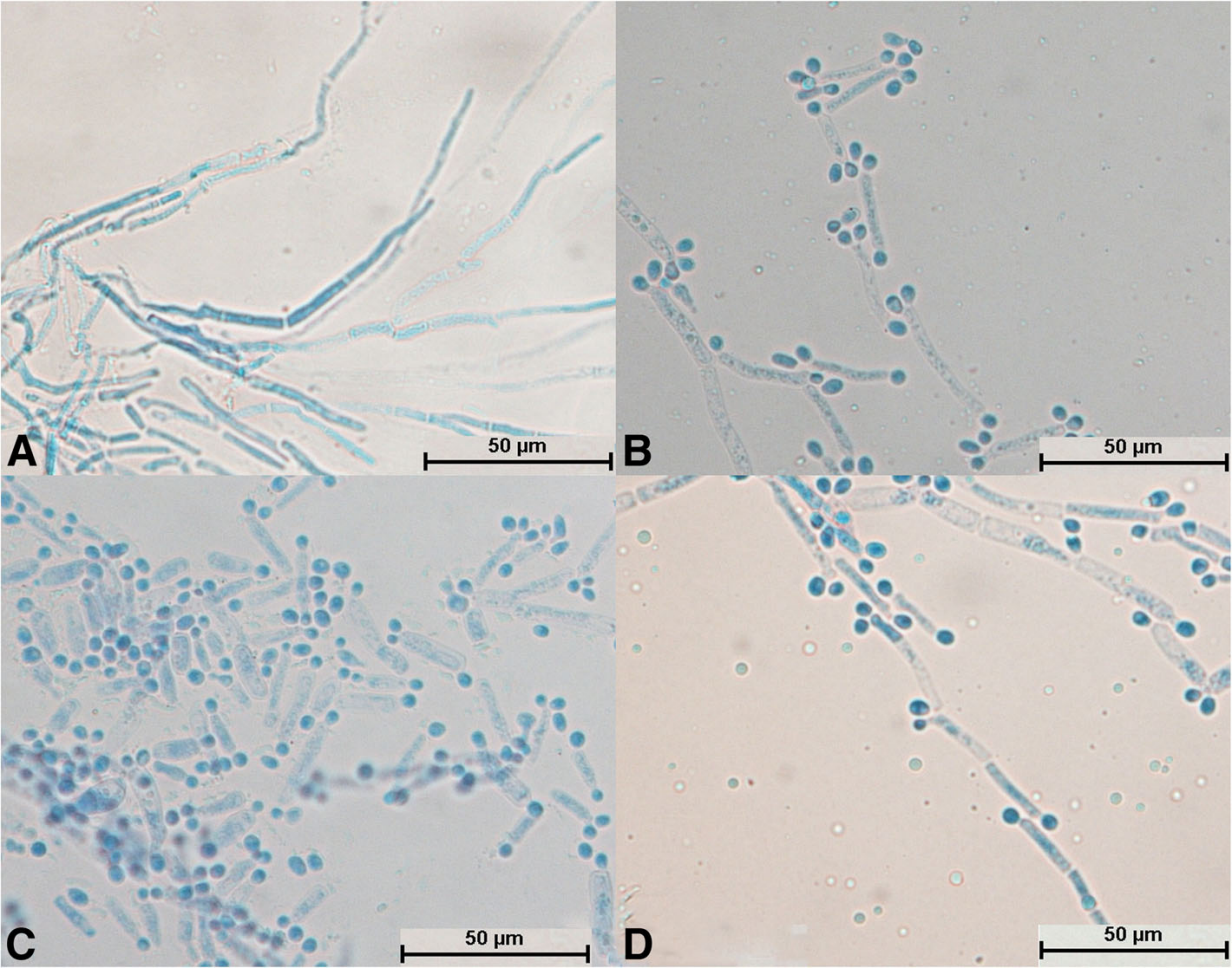
Morphological development process of *Trichosporon laibachii* from day 1 to day 4. **a** Some spores germinated and mycelium began to divide; **b**: Mycelium produced arthrospores; **c**: Mycelium was folded; **d**: Mature mycelium and arthrospores

The spores of *T. laibachii* grew into mycelium after cultivation for 24 h, and no spores divided independently (Fig. 4a). The mycelium spread radially (Fig. 4a) and was folded together (Fig. 4c), and differentiated into spores. Arthrospores were abundant, whereas spores were round and few in number (Fig. 4b, c, and d). The spores and mycelium were unevenly colored: the spores were darker, whereas the mycelium was lighter (Fig. 4b, c, and d). Hyphal folding was typical in structure.

**Fig. 5:**
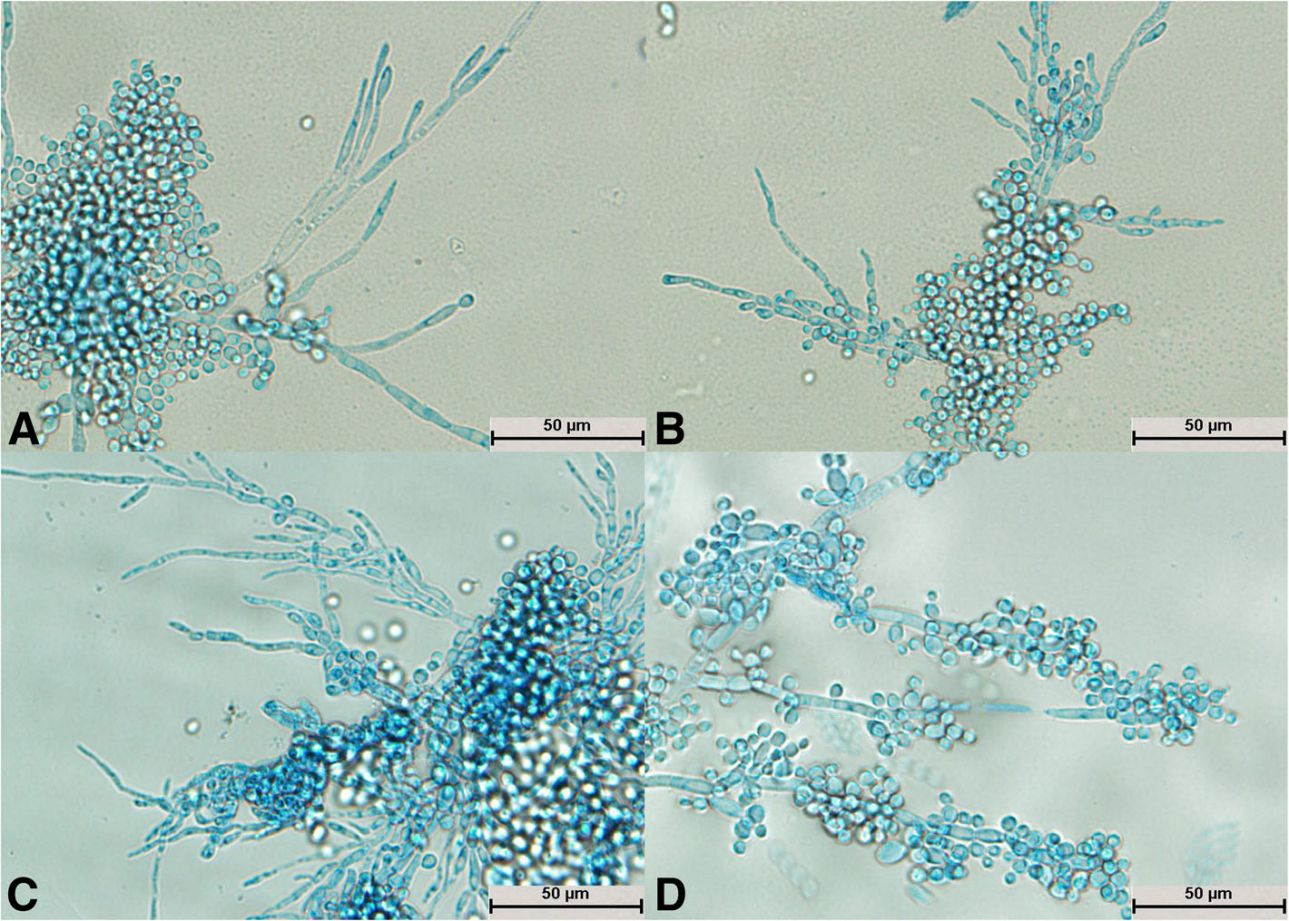
Morphological development process of *Trichosporon guehoae* from day 1 to day 4. **a** Mycelial buds formed new mycelium; **b**: New mycelium appeared at the point at which hyphae were segmented; **c**: A large number of botryoidal spores of *T. guehoae* were observed; **d**: New spores developed from arthrospores

The structural development of *T. guehoae* was completed after cultivation for 24 h (Fig. 5a). The mycelium spread radially but was scattered (Fig. 5a, b, c, and d). A large number of round spores were produced as grape-like clusters (Fig. 5a, b, and c). Hyphae and spores were evenly stained (Fig. 5a, b, c, and d). The mycelium grew from a segmented section to form a new mycelium (Fig. 5b), and the new spores were generated by arthrospores (Fig. 5d); this was a typical structure.

**Fig. 6:**
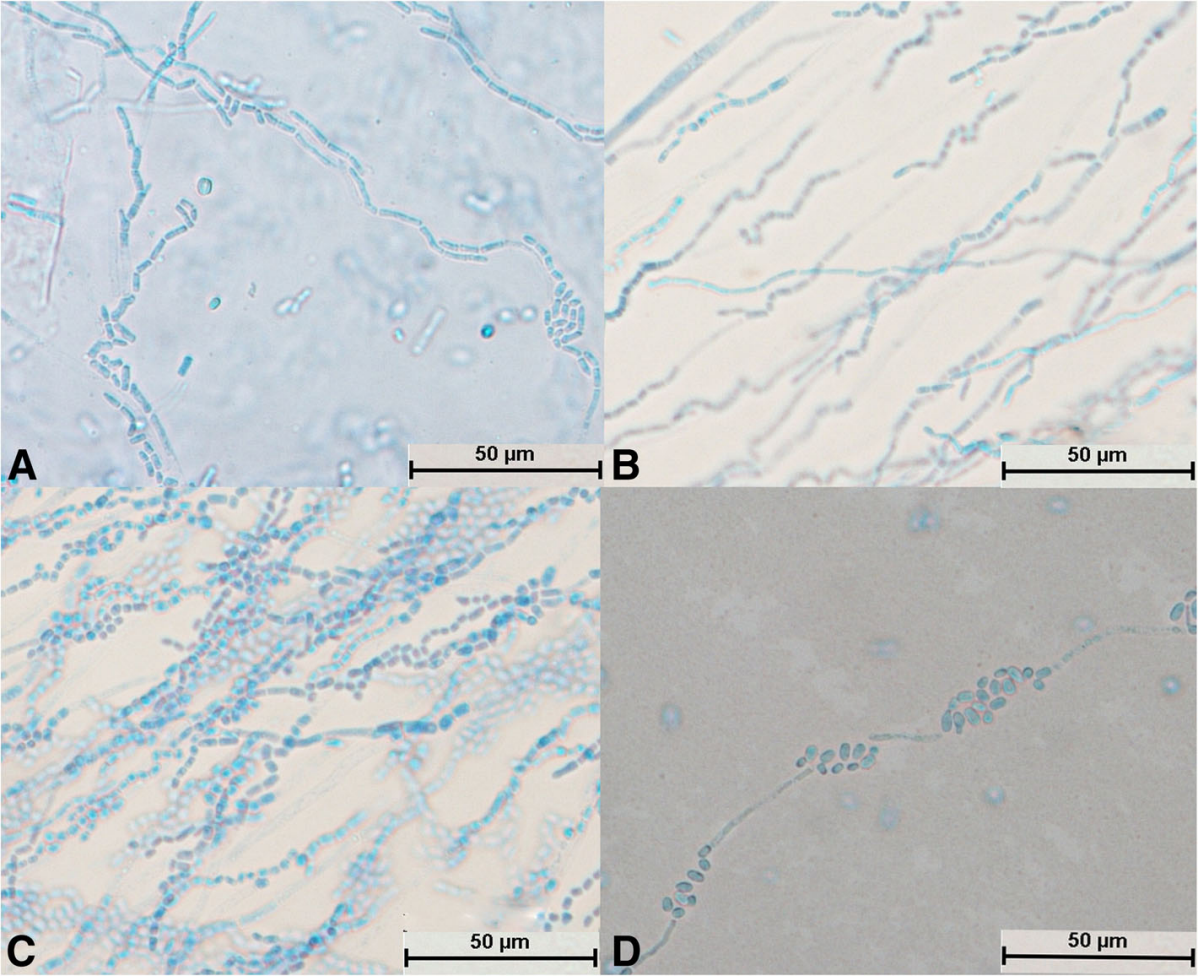
Morphological development process of *Trichosporon gracile* from day 1 to day 4. **a** and **b**: Hyphae were segmented; **c**: A large number of hyphae differentiated into spores, some of which were square; **d**: Mycelium divided into oval spores

After *T. gracile* was cultured for 24 h, a large number of hyphae appeared and became segments, and no spores could be seen (Fig. 6a). It could be seen that the mycelium differentiated into spores (Fig. 6b, c and d). A large number of spores were produced, which were mostly square (Fig. 6c) and became oval after reaching maturity (Fig. 6d). The mycelia and spores were evenly stained (Fig. 6c). Some hyphae did not divide into sections and differentiated into spores at intervals; this was a typical structure (Fig. 6d).

**Fig. 7:**
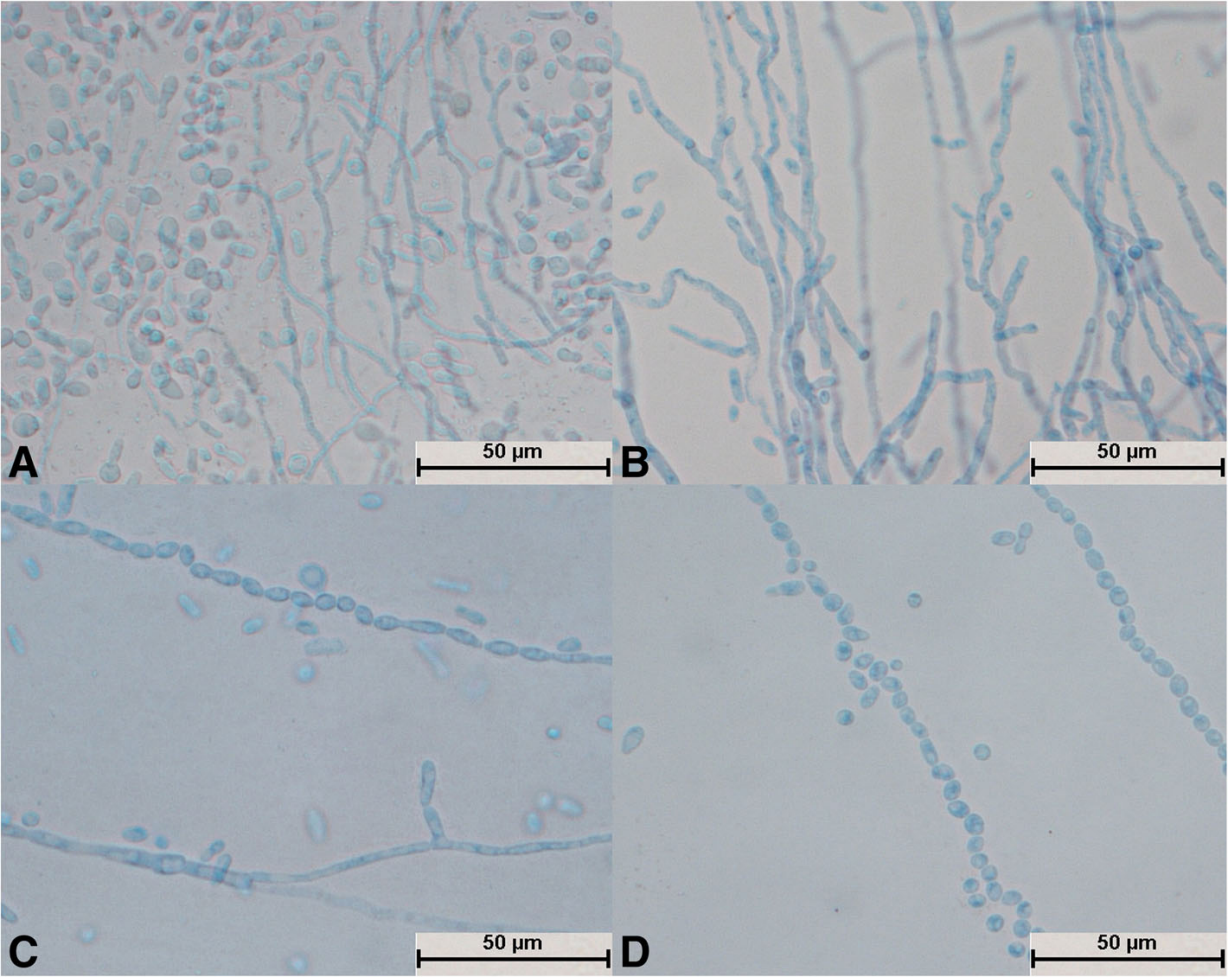
Morphological development process of *Trichosporon domesticum* from day 1 to day 4. **a** Spores expanded and sprouted; **b**: Mycelium had multiple branches and spindle-shaped buds; **c**: Mycelium began to differentiate into oval spores; **d**: Mycelium had completely differentiated into oval spores

After culture for 24 h, the spores of *T. domesticum* swelled to form mycelia and no spores divided independently (Fig. 7a). Hyphae were abundant and parallel to each other and had spindle-type buds (Fig. 7b). A large number of hyphae differentiated into spores (Fig. 7c and d). The number of spores was small with no arthrospores, and the spores were round (Fig. 7b, c, and d). The mycelia and spores were evenly stained (Fig. 7a, b, c, and d).

**Fig. 8:**
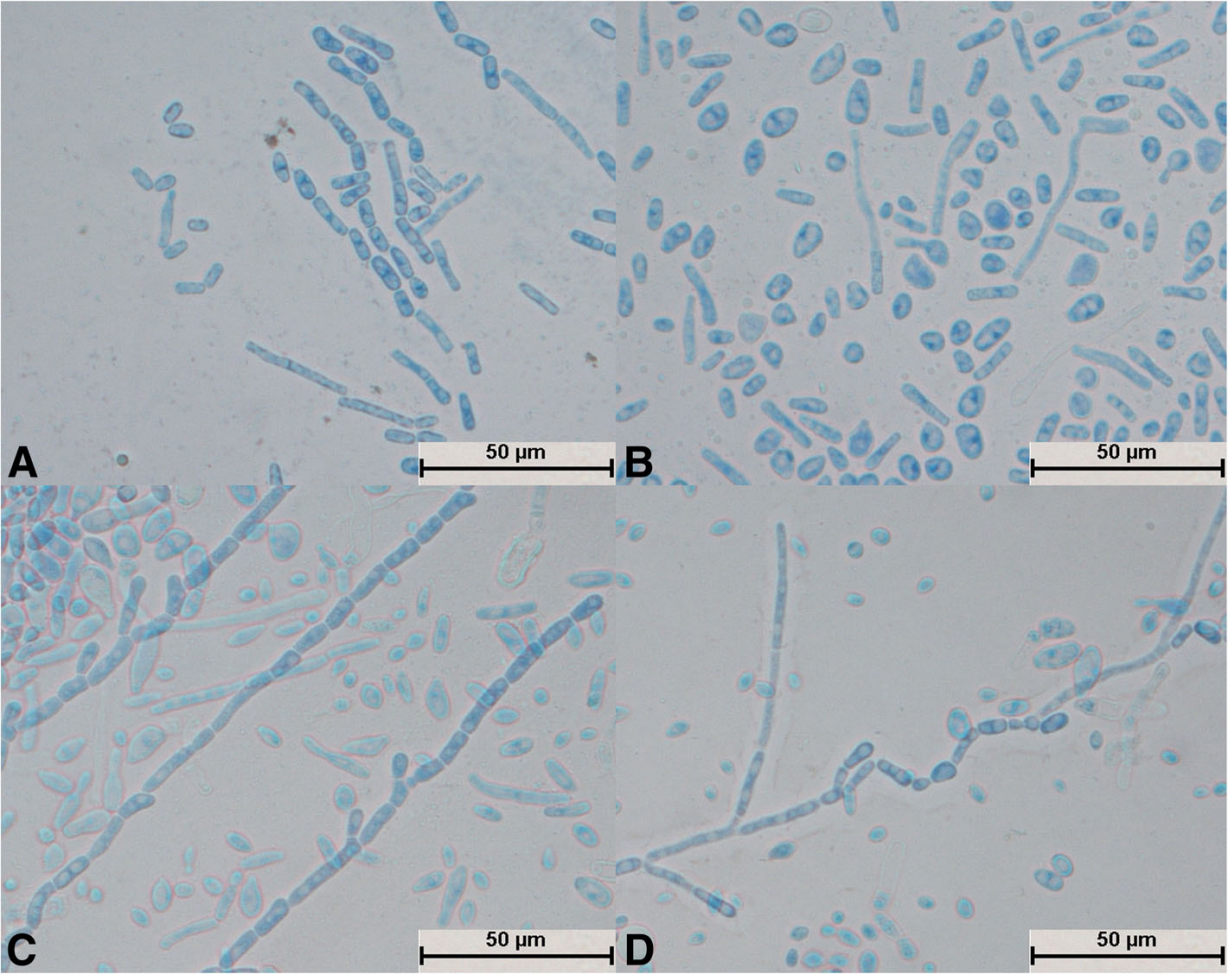
Morphological development process of *Trichosporon brassicae* from day 1 to day 4. **a** Spindle-shaped spores; **b**: Spores sprouted; **c**: Hyphae were segmented and spores were less oval; in the segmented hyphae, the arthrospores were fewer in number and spindle-shaped, and some spores developed into mycelium; **d**: Hyphae differentiated into spindle-shaped spores

After culture for 24 h, the spores of *T. brassicae* exhibited no significant changes (Fig. 8a), whereas after culture for 48 h the spores swelled and formed hyphae (Fig. 8b). No spores were found to divide independently (Fig. 8a and b). The mycelia became segmented and were distributed parallel to each other (Fig. 8c). It could be seen that the mycelium differentiated into spindle-shaped spores (Fig. 8d). The mycelium and spores were evenly stained (Fig. 8d), and there were few arthrospores (Fig. 8c).

**Fig. 9:**
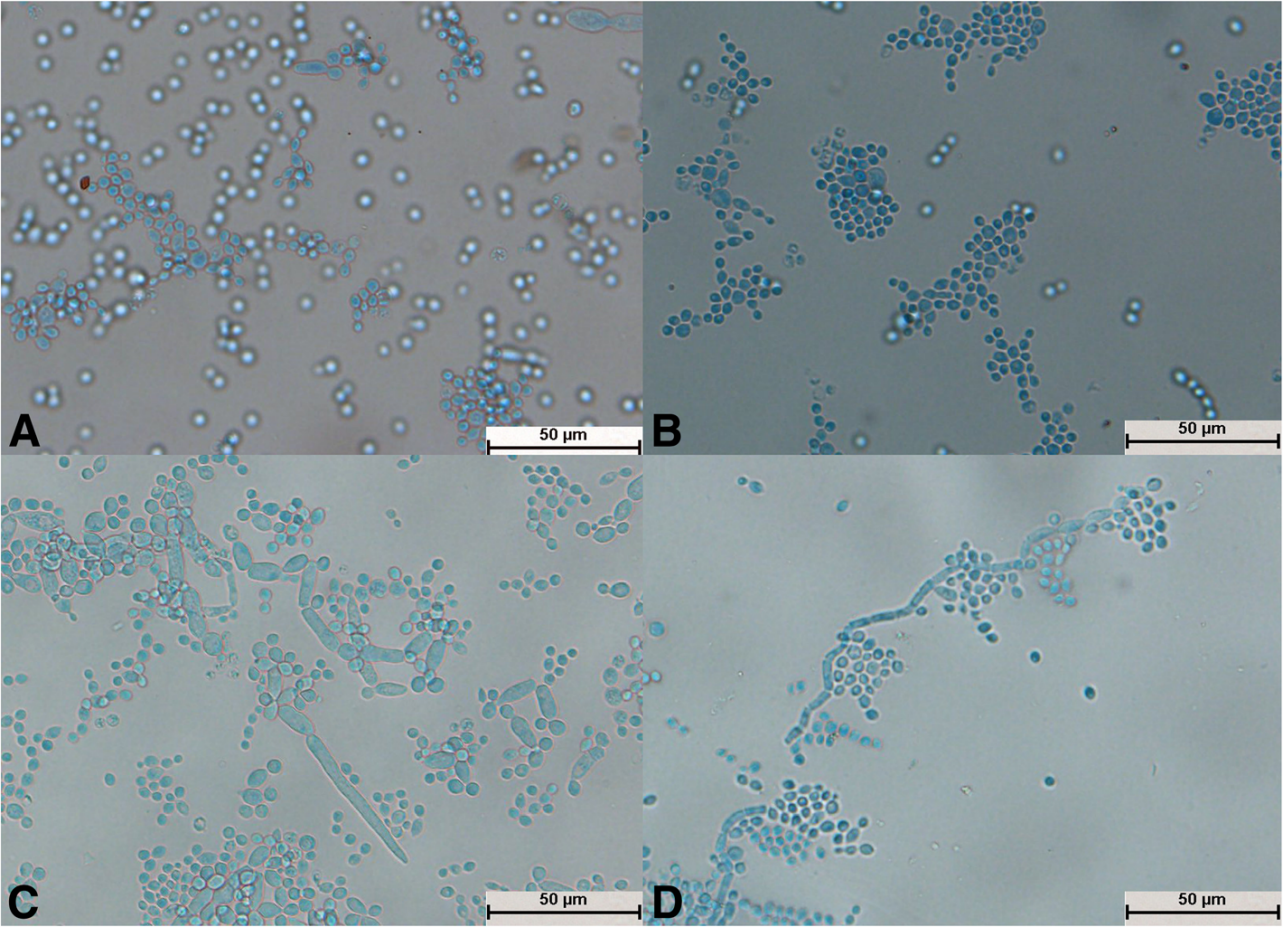
Morphological development process of *Trichosporon shinodae* from day 1 to day 4. **a** Spores swelled and budded; **b**: Spores divided; **c**: Spores formed thick and short hyphae; **d**: Mycelium produced a large number of arthrospores

After cultivation for 24 h, some spores of *T. shinodae* swelled (Fig. 9a), whereas other spores divided independently (Fig. 9b). The hyphae were short and sparse and grew very slowly (Fig. 9c). A large number of round arthrospores were produced (Fig. 9d). The spores and mycelium were evenly colored (Fig. 9c and d). Crude short mycelium was produced after culture for 72 h (Fig. 9c); this was a typical structure.

**Fig. 10:**
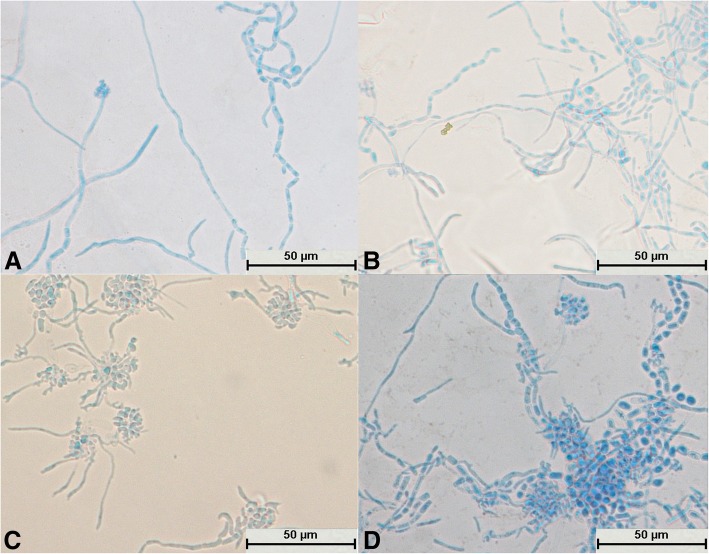
Morphological development process of *Trichosporon asteroides* from day 1 to day 4. **a** Tenuous mycelium, some of which began to become segmented; **b**: Mycelium differentiated into circular, oval, and spindle-shaped spores; **c**: The tail ends of mycelium produced a large number of small, aggregating spores and bifurcated; **d**: Mycelium differentiated into darker spores

After culture for 24 h, *T. asteroides* formed slender hyphae, and no spores divided independently (Fig. 10a). The mycelium was elongated (Fig. 10c) and could differentiate into spores (Fig. 10b and d). Spores on bifurcated mycelium aggregated into spheres (Fig. 10c); a large number of spores were produced, and the spores were round, oval, or spindle-shaped (Fig. 10b and d). The spores were not evenly pigmented and some were dark in color (Fig. 10d). The bifurcation of the mycelia and the aggregation of spores into spheres were typical structures (Fig. 10c).

**Fig. 11:**
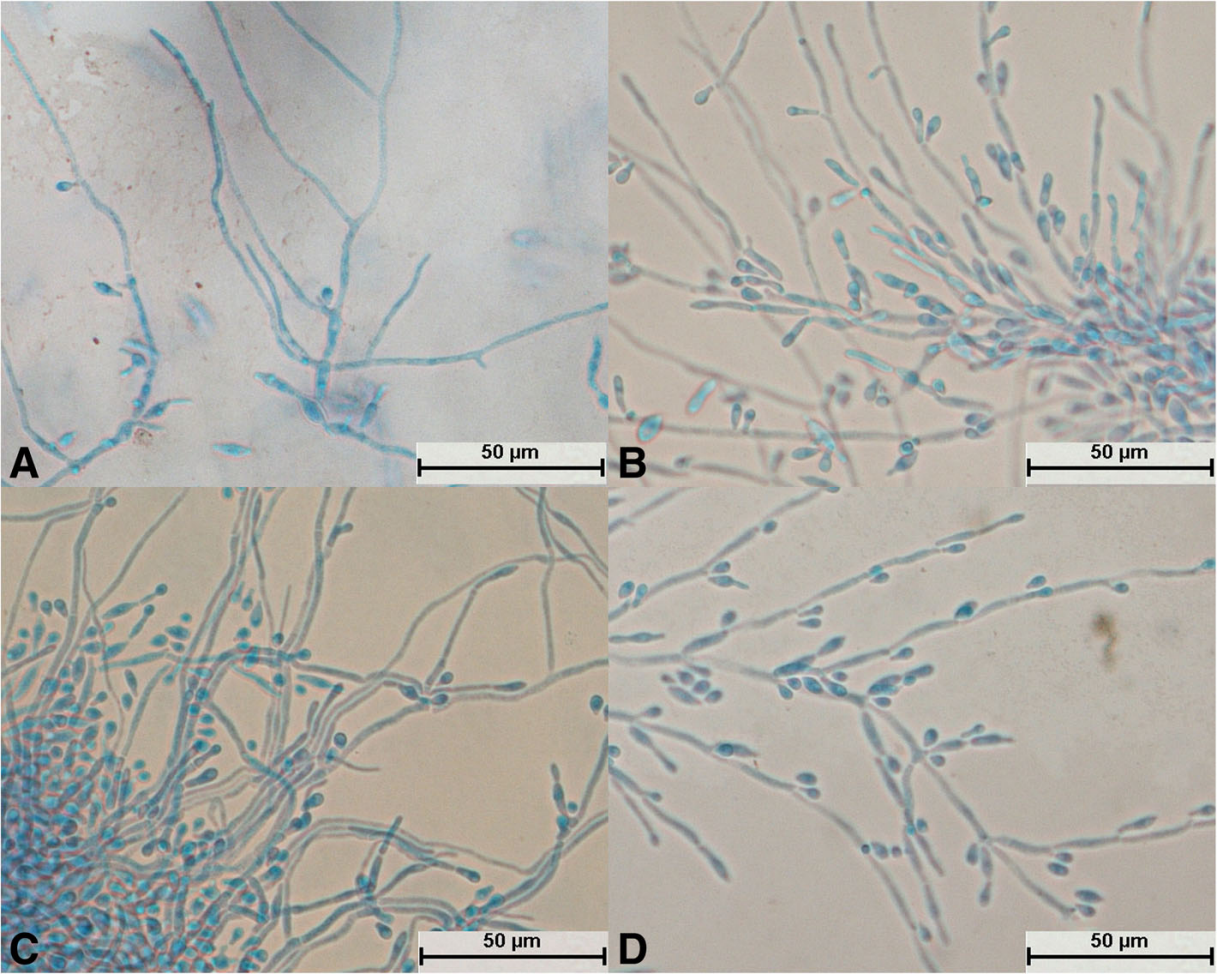
Morphological development process of *Trichosporon middelhovenii* from day 1 to day 4. **a** Hyphae were slender and did not divide; **b**: Hyphae produced fusiform arthrospores; **c**: Arthrospores budded to form new hyphae; **d**: Mature mycelium produced a large number of fusiform spores

After *T. middelhovenii* was cultivated for 24 h, hyphae were generated and no spores were found to divide independently (Fig. 11a). The hyphae were elongated (Fig. 11a and d), and their segments were small and indistinct (Fig. 11b and d). No hyphae differentiated into spores. Spores at bifurcations were spindle-shaped (Fig. 11a, b, c, and d). The spores were few in number and darker (Fig. 11c and d). Shuttle-type articular spores were characteristic structures (Fig. 11a, b, c, and d).

**Fig. 12:**
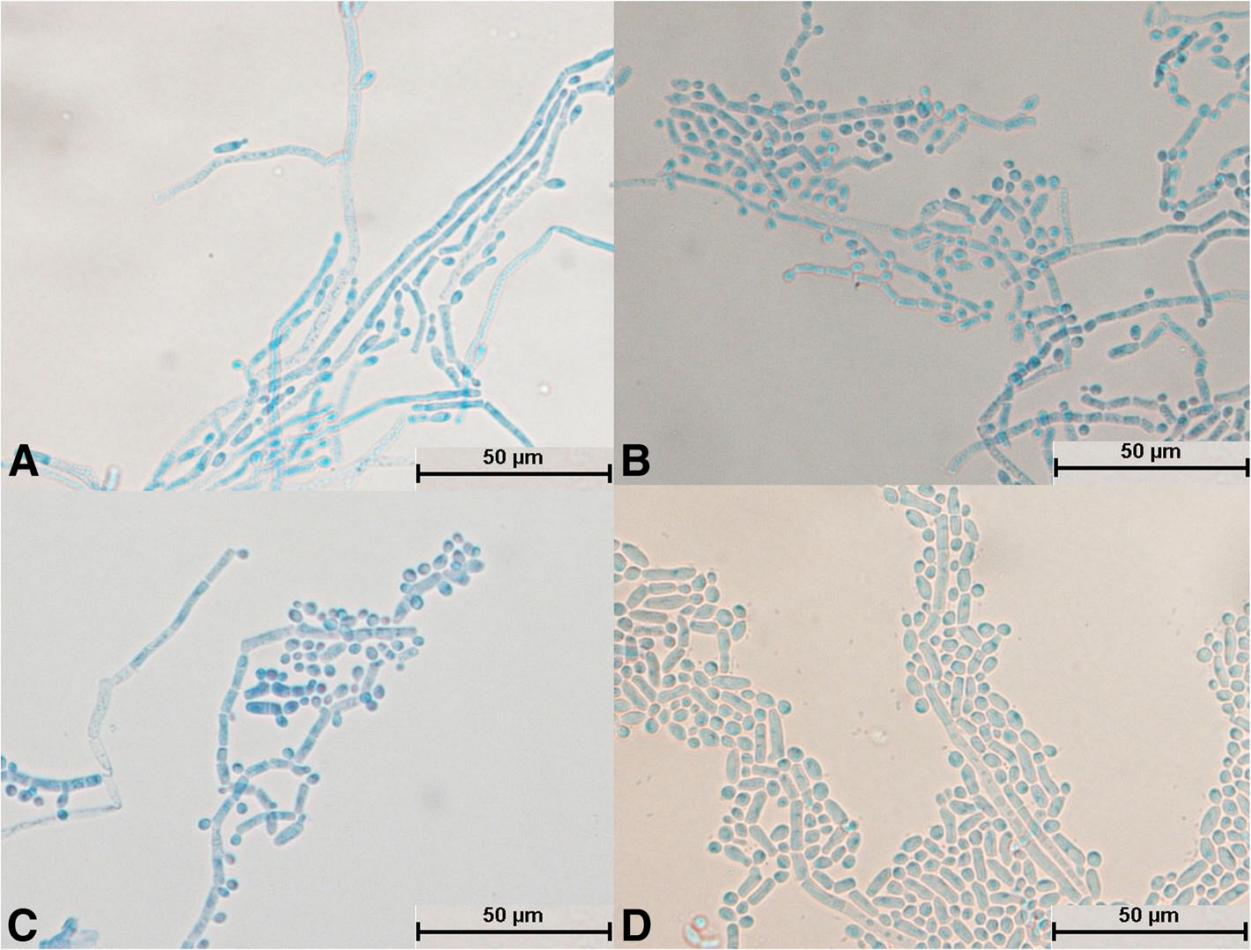
Morphological development process of *Trichosporon jirovecii* from day 1 to day 4. **a** Mycelium was slightly segmented and formed arthrospores; **b** and **c**: Mycelium formed a large number of circular arthrospores; **d**: Mycelium differentiated into spores

After *T. jirovecii* was cultivated for 24 h, hyphae appeared and no spores were seen to divide independently (Fig. 12a). The hyphae were segmented and radial (Fig. 12a) and differentiated into spores (Fig. 12d). Arthrospores were round and large in number (Fig. 12b); mycelia and spores were uniformly colored (Fig. 12d).

**Fig. 13:**
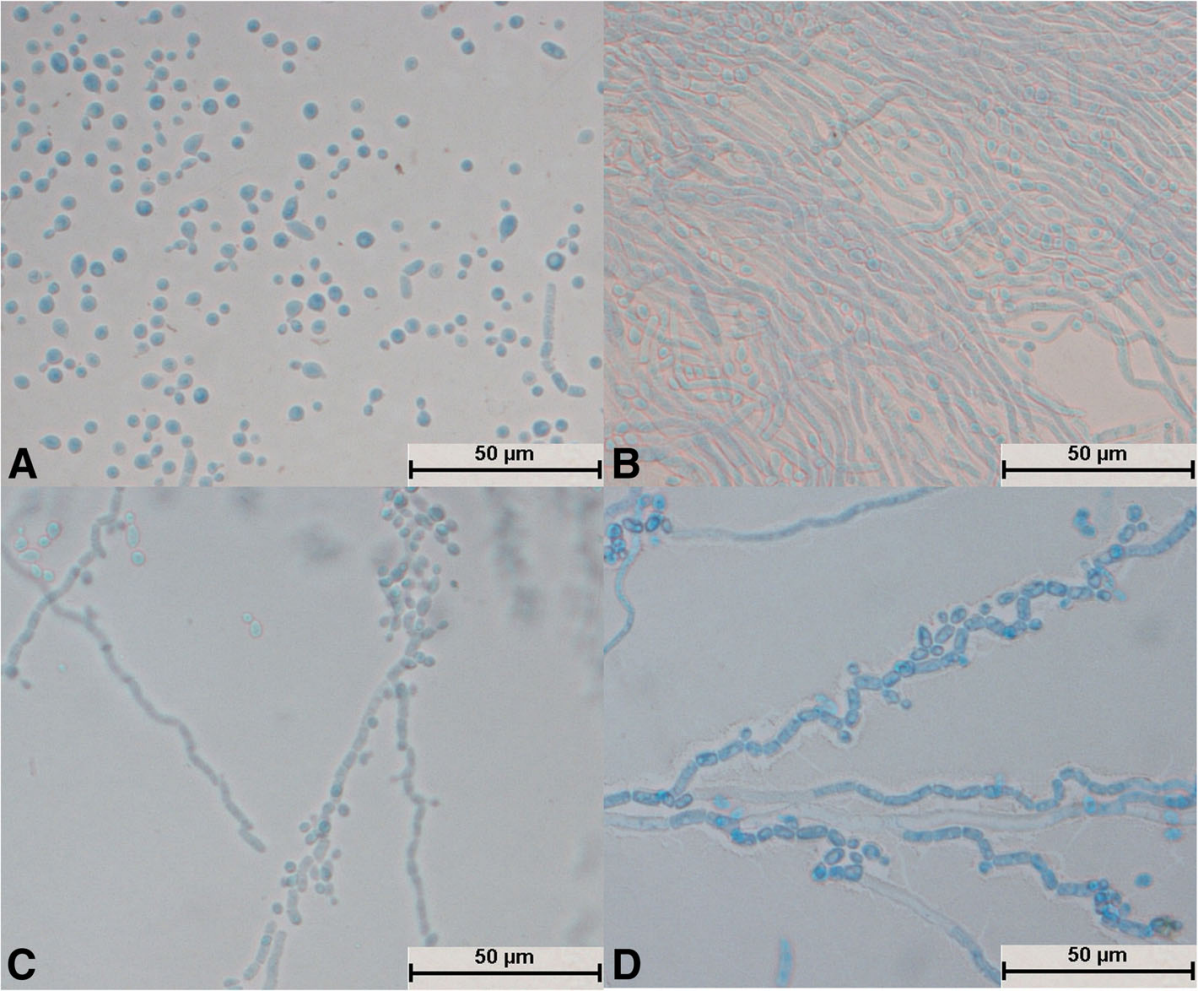
Morphological development process of *Trichosporon cutaneum* from day 1 to day 4. **a** Some spores expanded and sprouted, and some spores divided; **b**: Spores formed curved mycelium; **c**: Most mycelium was segmented, and the mycelium produced round arthrospores; **d**: Mycelium began to fold and differentiated into spores

After cultivation for 24 h, spores of *T. cutaneum* swelled and budded, and some spores divided independently (Fig. 13a). The mycelium was curved and segmented (Fig. 13b and c) and differentiated into spores (Fig. 13d). A large number of round arthrospores (Fig. 13c) were produced. The mycelium and spores were evenly stained (Fig. 13d), and curved mycelium was a typical structure (Fig. 13b and d).

### Pathogenicity

**Fig. 14:**
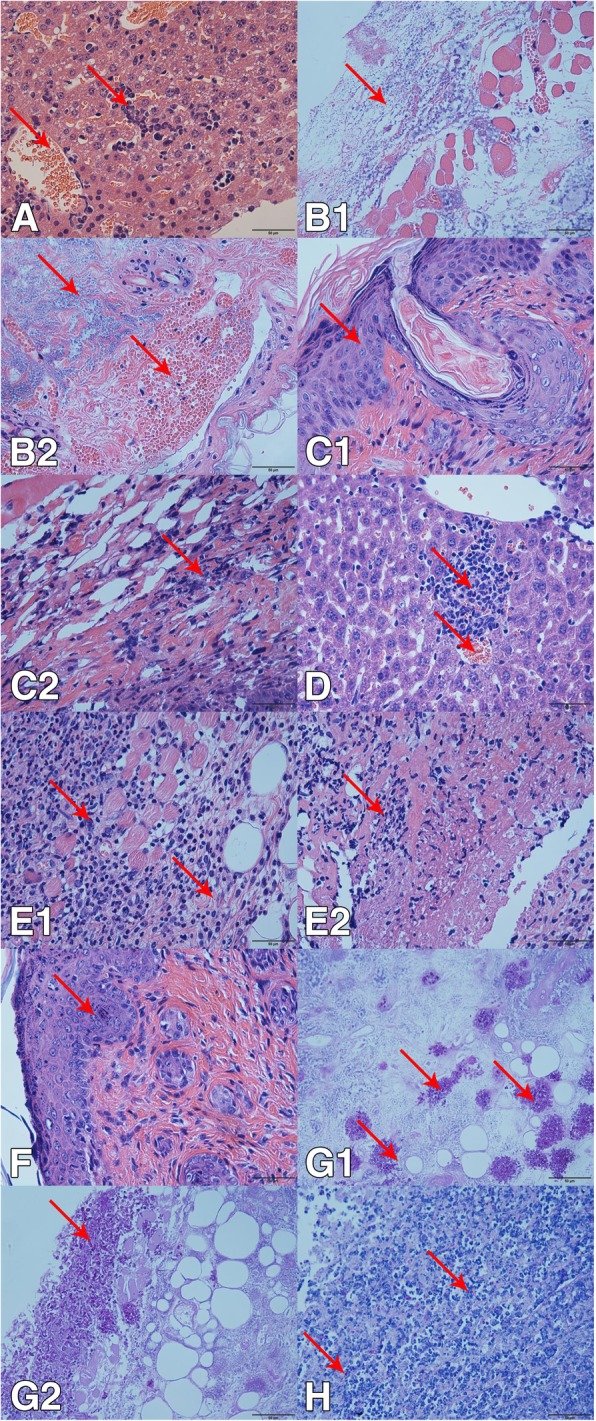
Pathological sections of tissue damaged by *Trichosporon asteroides* (JYZ1255) infection. **a**: Hepatocyte necrosis, hepatic sinusoidal congestion, and unclear hepatic cord structure; **b**1: Abscess of the dermis, necrosis of muscle tissue, and blood capillary congestion; **b**2: Epidermis with a large area of blood stasis and abscess; **c**1: Thickening of granular layer of skin and cuticle; **c**2: Infiltration of neutrophilic granulocytes into the epidermis; **d**: Hepatocyte necrosis and hepatic sinusoidal congestion; E1: A large number of neutrophils infiltrated into the reticular layer, muscle tissue, and dermis, and some cells are necrotic; E2: Osteonecrosis of the dermis and hyperplasia of connective tissue; F: Thickening of granular layer of skin and cuticle; G1: Spores stained with periodic acid/Schiff stain (PAS) in the reticular layer of the skin of a mouse in group B; G2: Spores stained with PAS in the dermis and spore germination in a mouse in group B; H: Spores stained with PAS in the dermis of a mouse in group E

The results of this study indicated that *Trichosporon* spp. mostly caused necrosis or swelling of hepatocytes and enlargement of the inter-hepatocyte space, and necrosis of hepatocytes mostly occurred near liver vessels. Subcutaneous injection of *Trichosporon* spp. caused lymphocyte infiltration into the skin, abscesses, and thickening of the stratum corneum. Mice that were inoculated via skin inunction had no obvious lesions, and most of them exhibited changes in the thickness of the stratum corneum, which in some cases resulted in subcutaneous abscesses. Tissues infected with *Trichosporon* spp. exhibited congestion easily both in skin and liver because of bleeding. Most of the *Trichosporon* spp. caused significant damage to the liver and skin, for example, *T. laibachii*, *T. brassicae*, *T. guehoae*, *T. asteroides*, *T. jirovecii*, *T. cutaneum*, *T. shinodae*, and *T. middelhovenii*. *T. asteroides*, *T. laibachii*, *T. brassicae*, *T. guehoae*, *T. cutaneum*, *T. shinodae*, and *T. middelhovenii* all produced spores in the skin infection model (Additional file 1: Figure S1, Additional file 2: Figure S2, Additional file 3: Figure S3, Additional file 4: Figure S4, Additional file 5: Figure S5, Additional file 6: Figure S6, Additional file 7: Figure S7, Additional file 8: Figure S8, Additional file 9: Figure S9, Additional file 10: Figure S10). In particular, *T. asteroides* gave rise to disseminated infections in the reticular layer of the skin (Fig. 14)G1 and budding in the dermis (Fig. 14G2). *T. gracile*, *T. moniliiforme*, and *T. domesticum* caused inconspicuous pathological changes, and hence their pathogenicity was weak.

## Discussion

### Interspecies identification of *Trichosporon* spp.

There have been reports on the isolation and identification of fungi from the body surface of the giant panda [4]. It was concluded that *Trichosporon* spp. were the dominant genus among skin flora on the giant panda [4]. Recently, there have been many reports on infections by *T. asahii* [21–26], but few mentions of other *Trichosporon* spp.[9, 27]. However, there have been reports that some animals are susceptible to rare *Trichosporon* spp.[27–29]. Because the phylogenetic relationship between *Trichosporon* spp. was very close, it was impossible to distinguish the different species of *Trichosporon* spp. according to the ITS region or D1/D2 domain in every case [30]. The sequence similarity between the ITS regions of *T. asahii* and *T. asteroides*, in which only two or three bases are different, is 99–99.3%, and *T. montevideense* and *T. domesticum* have identical ITS regions [31]. Scorzetti et al. found that the differences between the 28 s rDNA D1/D2 domains of different *Trichosporon* spp. are greater than those between the corresponding ITS regions. The ITS regions of *T. laibachii* and *T. multisporum* are identical, and seven bases are different in the D1/D2 domains. Two bases are different in the D1/D2 domains of *T. montevideense* and *T. domesticum* [32]. Guo amplified all three loci (ITS, D1/D2, and IGS1) and constructed a phylogenetic tree for the ITS region and D1/D2 domain and a separate phylogenetic tree for the IGS1 region. Both trees could completely distinguish the *Trichosporon* spp.[13]. In this study, seven strains could not be identified by their IGS1 regions because of the lack of sequence information for IGS1 in the NCBI database. Hence, we used the joint contribution of the ITS region and D1/D2 domain, which we compared with the phylogenetic tree for IGS1. It was found that the clades of the phylogenetic trees were basically identical and authenticated each other, so that all 29 strains could be identified completely.

### Pathogenicity of dominant *Trichosporon* spp. isolated from pandas

*T. asteroides* and *T. jirovecii* were the dominant *Trichosporon* spp. that were isolated from the giant panda samples, and these species are widely present in giant pandas [4]. Their pathogenicity has a great influence on the health of giant pandas [4]. Especially, *T. asteroides* showed high pathogenicity because it caused disseminated infections in the reticular layer of the skin. This is consistent with the results of Chagas-Neto’s report [15]. *T. jirovecii* genotype 1 has been isolated from the human body [13], but its pathogenicity was unknown. Thus far, there have been few reports on *T. jirovecii*: Malgorzata et al. reported one case of mixed respiratory infection in a dog caused by *T. jirovecii* and *Rhodotorula* [33, 34], and Nardoni reported one case of back infection in a tortoise caused by *T. jirovecii* [10]. In the present study, four strains of *T. laibachii* (JYZ3252, JYZ921, JYZ321, and JYZ912) and one strain of *T. moniliiforme* (JYZ372) were identified as having new genotypes. Their pathogenicity remains to be confirmed by future studies.

### Genotyping of *Trichosporon* spp.

At present, IGS1 sequence analysis is generally used for genotyping *Trichosporon* spp. For example, Chagas-Neto et al. completed the genotyping of 14 strains of *T. asahii* by IGS sequence analysis [15], whereas Guo completed the genotyping of 39 strains of *T. asahii* [12]. The main target in the genotyping of *Trichosporon* spp. has been *T. asahii*, and the genotyping of other *Trichosporon* spp. has been rare. In this study, among all the isolates only *T. jirovecii* had been assigned to two genotypes, and the other species had not been studied [13]. The main reason was that the identification of *Trichosporon* spp. is difficult and sequence information for IGS1 is scarce. In this study, only preliminary genotyping was performed for *Trichosporon* spp., and further research will rely on improvements in sequence information for IGS1in *Trichosporon* spp.

### Morphological development process of *Trichosporon* spp.

There have been few studies on the morphology of *Trichosporon* spp. In 2005, Li et al. performed ITS-PCR detection and morphological and susceptibility testing on six *Trichosporon* spp. [16]. The colonies of different *Trichosporon* spp. were similar, but the morphologies of their mycelia and spores were significantly different. The structure of the mycelium was not destroyed, and the test results were credible. The morphology of the mycelia of *T. domesticum* was very similar to that in this study. The morphological development process of *Trichosporon* spp. was significantly different, and the majority of *Trichosporon* spp. had a typical structure: for example, septal differentiation of the mycelium in *T. gracile* (Fig. 8d); short thick mycelium during the development of *T. shinodae* (Fig. 11c); elongation and bifurcation of the mycelium and the aggregation of spores into spheres in *T. asteroides* (as shown in Fig. 10c); and a spindle-type articular spore structure in *T. middelhovenii* (Fig. 13a, b, c, and d). The above results proved that the morphological development process and typical structure have great significance as references for morphological identification.

From the point of view of the development and sporulation of mycelia, *Trichosporon* is an intermediate genus between molds and yeasts. Its mycelia can differentiate into a large number of spores like yeasts and also produce conidia like molds. Spores in the early stages of development can either bud like hyphae or divide like those of yeasts. Colonies of some *Trichosporon* spp. resemble yeasts in being milky, oily, and reflective, whereas colonies of some *Trichosporon* spp. have a radiate texture similar to that of molds [16]. *Trichosporon* might represent an intermediate genus in the evolution of yeasts into molds. In the study of the morphology of *Trichosporon* spp., they should be regarded as molds in order to observe their sporulation and mycelial structure.

### Pathological changes in *Trichosporon* spp. infections

Different *Trichosporon* spp. cause similar pathological lesions on the skin and liver. *T. asahii* caused hepatic sinusoidal dilatation, mild to moderate dilatation of small blood vessels, hyperemia, neutrophil-based focal infiltration of inflammatory cells, and proliferation or degeneration of hepatocytes [35]. *T. dermatis* caused hepatic sinusoidal dilatation and congestion, swelling, degeneration, or necrosis of hepatocytes, and hyperplasia of Kupffer cells [36]. These lesions were similar to the pathological changes in the liver observed in this study. In the literature there are few mentions of skin lesions, subcutaneous abscesses, and bruises that were caused by *T. dermatis*.

However, most of the *Trichosporon* spp. identified in this study could cause skin lymphocyte infiltration, abscesses, and thickening of the stratum corneum. The pathological changes were significantly different between the groups treated by subcutaneous injection and skin inunction. These conclusions were similar to those of a study that was reported in China for the first time in 2010[37]. The skin damage caused in the group treated by skin inunction was lighter, and only *T. laibachii* and *T. asteroides* caused obvious pathological changes, which might be related to the uncontrollable amount of the spore coating and the pathogenicity of the *Trichosporon* spp. themselves. The spores developed a strong tendency to form mycelium, and the process of formation of mycelium could cause mechanical damage, which might be a reason for these observations.

### Pathogenicity of the *Trichosporon* spp.

Except for *T. moniliiforme*, *T. domesticum*, and *T. gracile*, all the *Trichosporon* spp. in this study caused significant damage to the liver and skin in healthy mice (Additional file 1 Figure S1, Additional file 2 Figure S2, Additional file 3 Figure S3, Additional file 4 Figure S4, Additional file 5 Figure S5, Additional file 6 Figure S6, Additional file 7 Figure S7, Additional file 8 Figure S8, Additional file 9 Figure S9, Additional file 10 Figure S10). In most cases spores stained with PAS could be observed in skin sections. *T. brassicae*, *T. guehoae*, *T. middelhovenii*, and *T. shinodae* were found for the first time to be pathogenic forms of *Trichosporon* that could provoke obvious lesions in immunosuppressed and non-immunosuppressed groups. Hitherto, reports of the pathogenicity of these four *Trichosporon* spp. had not been found. This might be related to difficulties in the identification of *Trichosporon* spp. and differences in pathogenicity caused by the differences between strains.

In this study, *T. asteroides* (JYZ1255) exhibited strong pathogenicity. The infected tissue was extensively congested, and there was a large area of ​​abscess. Two mice in group A died 2 days after being inoculated with a suspension of *T. asteroides*. There have been many reports on *T. asteroides*, which is one of the main pathogens involved in trichosporosis in humans and is a dominant strain among fungi on the body surface of the giant panda. *T. asteroides* caused purulent keratitis [38] and was also isolated from the blood of patients with disseminated trichosporosis [13, 15]. *T. asteroides* gave rise to obvious disseminated infections in the reticular layer of the skin (Fig. 14G1) and budding in the dermis (Fig. 14G2). The infections might cause damage or even be life-threatening to immunocompromised giant pandas. *T. jirovecii* (JYZA10) was identified as having genotype 1 in previous studies (Fig. 1) and was significantly more pathogenic in immunosuppressed mice than in non-immunosuppressed mice and tissue. The degree of damage was significantly lower than that caused by *T. asteroides*, and it was inferred that genotype 1 of *T. jirovecii* was opportunistically pathogenic. Four strains of *T. laibachii* were identified as having new genotypes by phylogenetic analysis of the IGS1 sequence (Fig. 2). Although there have currently been no reported cases of infection involving *T. laibachii*, *T. laibachii* (JYZ3252) caused skin ulceration in mice (Fig. 4). This lesion demonstrated that the new genotype of *T. laibachii* is more pathogenic.

The pathogenicity test only studied the effects of *Trichosporon* spp. on the skin and liver, and most of the isolated *Trichosporon* spp. caused more severe damage to skin than to the liver. For example, *T. brassicae* (JYZ1253) and the reference strain, which was isolated from rancid milk, had the same genotype, but the isolated strain caused severe inflammatory reactions in skin tissue and skin necrosis. *T. asteroides* (JYZ1255) and the reference strain, which was isolated from human blood, had the same genotype, but the isolated strain caused disseminated infections in the reticular layer of the skin. It was assumed that the pathogenicity of *Trichosporon* spp. is related to their parasitic environment and that *Trichosporon* spp. that are isolated from the skin surface cause more pronounced damage to the skin.

## Conclusions

We can concluded that combination of ITS, D1/D2, and IGS1 loci can effectively identify the genotype of *Trichosporon* spp. The morphological development process and typical structure of *Trichosporon* molds type have great significance as references for morphological identification.

## Additional files

Additional file 1: **Figure S1.** Pathological sections of tissue damaged by *Trichosporon gracile* (JYZ1291) infection. A: Central venous congestion of the liver, interstitial widening, and a small amount of lymphocyte proliferation (400×); B: Thickening of the cuticle of the skin and a small amount of lymphocyte proliferation (400×); C: Mild congestion in the reticular layer (400×); D: Central venous congestion of the liver, hepatic sinusoidal congestion, swelling of hepatocytes and proliferation of lymphocytes (400×); E: Normal structure (400×); F: Thickening of the cuticle and granular layer (400×). (PDF 488 kb)

Additional file 2: **Figure S2**. Pathological sections of tissue damaged by *Trichosporon brassicae* (JYZ1253) infection. A: Central venous congestion of the liver, hepatic sinusoidal congestion, infiltration of lymphocytes, and interstitial widening (400×); B1: Congestion in the dermal papillary layer and thickening of the cuticle of the skin (400×); B2: Massive cell necrosis of the reticular layer of the skin, local coagulation necrosis, and a large amount of lymphocyte infiltration (400×); C: Thickening of the cuticle of the skin and infiltration of a few lymphocytes (400×); D: Central venous congestion and interstitial widening of the liver (400×); E: Necrosis of the reticular cells of the skin, local coagulation necrosis, and infiltration of a large number of lymphocytes (400×); F: Normal structure (400×); G: Spore stained with PAS in a lesion of the dermis of a mouse in group B (400×). (PDF 674 kb)

Additional file 3: **Figure S3.** Pathological sections of tissue damaged by *Trichosporon domesticum* (JYZ983) infection. A: Local necrosis and swelling of hepatocytes and infiltration of a small number of lymphocytes (400×); B: Thickening of the cuticle of the skin (400×); C: Normal structure (400×); D: Mild interstitial widening and swelling of hepatocytes (400×); E: Small amount of lymphocyte proliferation (400×); F: Normal structure (400×). (PDF 451 kb)

Additional file 4: **Figure S4.** Pathological sections of tissue damaged by *Trichosporon guehoae* (JYZ1221) infection. A: Hepatocyte necrosis, lymphocyte infiltration, hepatocyte swelling, and unclear hepatic cord structure (400×); B: Thickening of the cuticle of the skin and infiltration of reticular lymphocytes (400×); C: Normal structure (400×); D: Local necrosis of hepatocytes and diffuse congestion (400×); E: Necrosis of skin cells; F: Normal structure (400×); G: Spore stained with PAS in a lesion of the dermis of a mouse in group B (400×). (PDF 593 kb)

Additional file 5: **Figure S5.** Pathological sections of tissue damaged by *Trichosporon jirovecii* (JYZA10) infection. A: Central venous congestion of the liver, mild lymphocyte infiltration, and hepatocyte swelling (400×); B1: Thickening of the cuticle and necrosis of skin cells (200×); B2: Necrosis of reticular cell (400×); C: Normal skin structure (400×); D: Diffuse congestion of the liver and interstitial widening (400×); E: Thickening of the cuticle, infiltration of inflammatory cells, and local congestion of the reticular layer (400×); F: Normal skin structure (400×). (PDF 563 kb)

Additional file 6: **Figure S6.** Pathological sections of tissue damaged by *Trichosporon cutaneum* (JYZ030202) infection. A: Central venous congestion of the liver, necrosis of liver cells around the veins, and infiltration of lymphocytes (400×); B1: Necrosis of cells in the papillary layer and infiltration of lymphocytes (400×); B2: Necrosis of reticular cells in the skin and infiltration of lymphocytes (400×); C: Normal skin structure (400×); D: Central venous congestion of the liver, hepatic sinusoidal congestion, swelling of liver cells, and disorder of the hepatic cord (400×); E: Necrosis of reticular cells in the skin and proliferation of lymphocytes (400×); F: Normal skin structure (400×); G: Spore stained with PAS in a lesion in the dermis of a mouse in group B (400×); H: Spore stained with PAS in a lesion in the dermis of a mouse in group E (400×). (PDF 721 kb)

Additional file 7: **Figure S7.** Pathological sections of tissue damaged by *Trichosporon shinodae* (JYZ1223) infection. A: Central venous congestion of the liver, local necrosis of hepatocytes, and mild lymphocyte infiltration (400×); B: Thickening of the cuticle and granular layer, local necrosis of cells in the reticular layer, and proliferation of lymphocytes (400×); C: Normal skin structure (400×); D: Central venous congestion of the liver, hemorrhage of the hepatic sinusoids, swelling of liver cells, and infiltration of a small number of lymphocytes (400×); E: Slight thickening of the cuticle of the skin, local necrosis of reticular cells, and infiltration of lymphocytes (400×); F: Normal skin structure (400×); G: Spore stained with PAS in the dermis of a mouse in group B (400×). (PDF 610 kb)

Additional file 8: **Figure S8.** Pathological sections of tissue damaged by *Trichosporon middelhovenii* (JYZ12922) infection. A: Diffuse congestion, venous congestion, hepatocyte swelling, unclear structure of the hepatic cord, and proliferation of lymphocytes (400×); B: Coagulative necrosis of reticular cells in the skin and proliferation of lymphocytes (400×); C: Thickening of the cuticle of the skin and proliferation of reticular lymphocytes (400×); D: Central venous congestion and interstitial widening (400×); E: Coagulative necrosis of skin cells, unclear structure of skin tissue, and proliferation of lymphocytes (400×); F: Thickening of the cuticle of the skin (400×); G: Spore stained with PAS in the dermis of a mouse in group C (400×); H: Spore stained with PAS in the dermis of a mouse in group B (400×). (PDF 679 kb)

Additional file 9: **Figure S9.** Pathological sections of tissue damaged by *Trichosporon moniliiforme* (JYZ932) infection. A: Hepatocyte necrosis (400×); B: Thickening of the cuticle (400×); C: Basically normal structure of skin(400×); D: Proliferation of hepatocytes in the liver (400×); E: Proliferation of lymphocytes in the skin (400×); F: Basically normal structure of skin (400×). (PDF 430 kb)

Additional file 10: **Figure S10.** Pathological sections of tissue damaged by *Trichosporon laibachii* (JYZ3252) infection. A: Central venous congestion of the liver, necrosis and swelling of hepatocytes, and unclear hepatic cord structure (400×); B1: Thickening of the cuticle of the skin (400×); B2: Mild necrosis of cells in the reticular layer (400×); C: Necrosis of skin cells, punctate infiltration of lymphocytes, and thickening of the granular layer (200×); D: Central venous congestion of the liver, hepatocyte necrosis, local infiltration of inflammatory cells, hepatocyte swelling, and unclear hepatic cord structure (400×); E: Thickening of the skin and local congestion (400×); F: Thickening of the cuticle (200×); G: Spore stained with PAS in a lesion in the dermis of a mouse in group C. (PDF 648 kb)
