# Supplementary figures and images for: Identification, genotyping, and pathogenicity of Trichosporon spp. Isolated from Giant pandas (Ailuropoda melanoleuca)
Source: BMC Microbiol. 2019 May 28;19:113. doi: 10.1186/s12866-019-1486-7 (PMC6540556; doi:10.1186/s12866-019-1486-7)

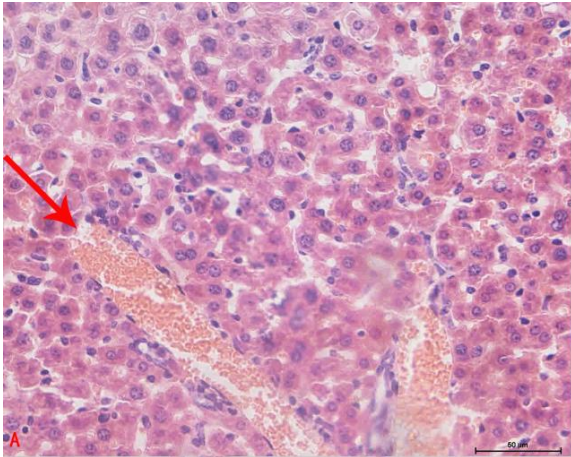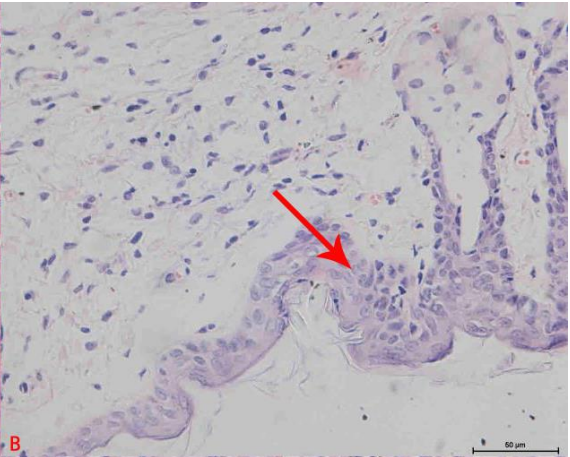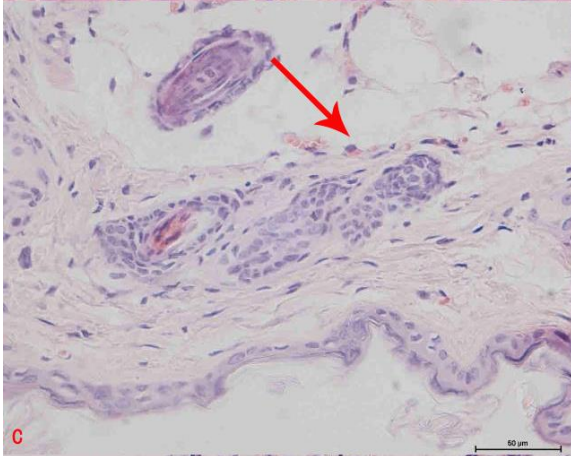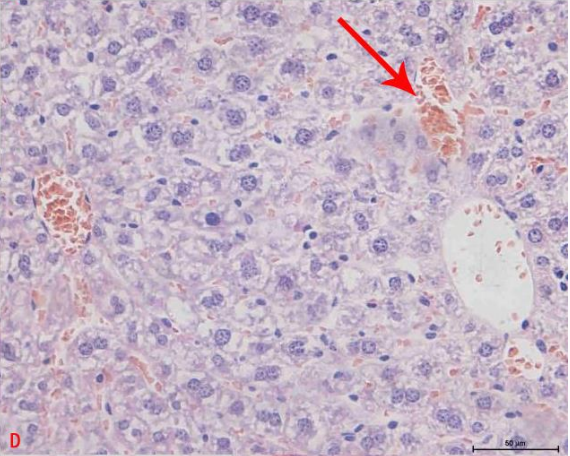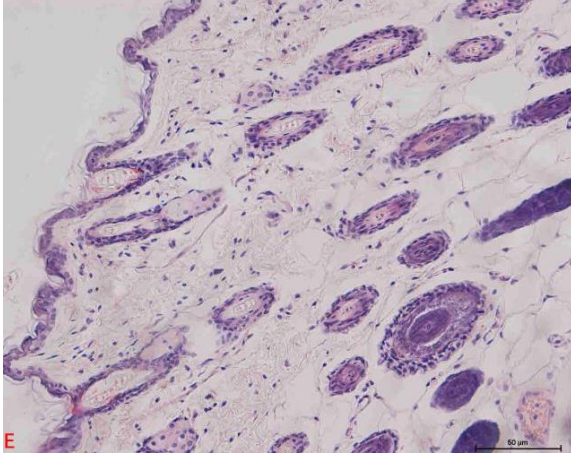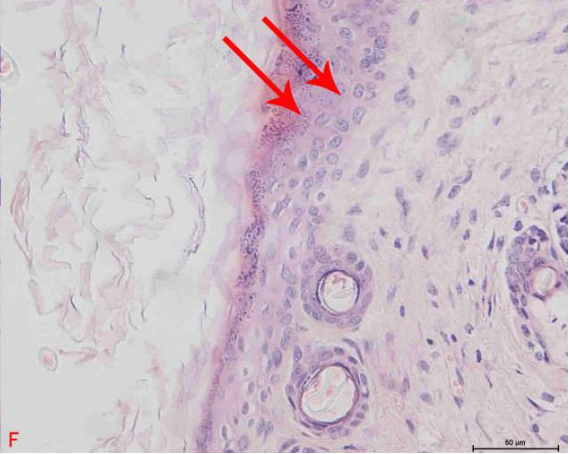

Supplement: Supplementary file 1 — Figure S1. Pathological sections of tissue damaged by Trichosporon gracile (JYZ1291) infection. A: Central venous congestion of the liver, interstitial widening, and a small amount of lymphocyte proliferation (400×); B: Thickening of the cuticle of the skin and a small amount of lymphocyte proliferation (400×); C: Mild congestion in the reticular layer (400×); D: Central venous congestion of the liver, hepatic sinusoidal congestion, swelling of hepatocytes and proliferation of lymphocytes (400×); E: Normal structure (400×); F: Thickening of the cuticle and granular layer (400×). (PDF 488 kb) [file 12866_2019_1486_MOESM1_ESM.pdf]

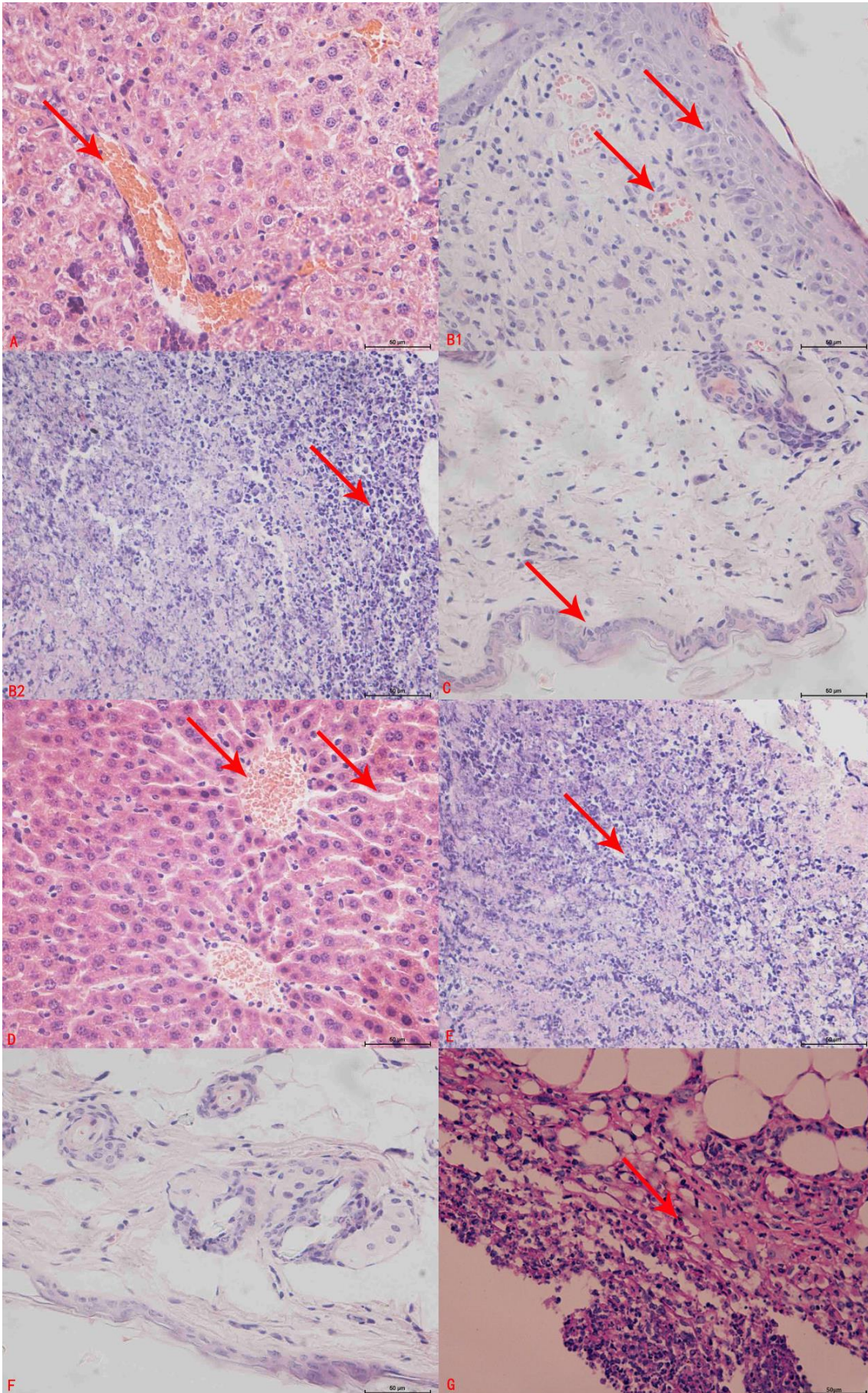

Supplement: Supplementary file 2 — Figure S2. Pathological sections of tissue damaged by Trichosporon brassicae (JYZ1253) infection. A: Central venous congestion of the liver, hepatic sinusoidal congestion, infiltration of lymphocytes, and interstitial widening (400×); B1: Congestion in the dermal papillary layer and thickening of the cuticle of the skin (400×); B2: Massive cell necrosis of the reticular layer of the skin, local coagulation necrosis, and a large amount of lymphocyte infiltration (400×); C: Thickening of the cuticle of the skin and infiltration of a few lymphocytes (400×); D: Central venous congestion and interstitial widening of the liver (400×); E: Necrosis of the reticular cells of the skin, local coagulation necrosis, and infiltration of a large number of lymphocytes (400×); F: Normal structure (400×); G: Spore stained with PAS in a lesion of the dermis of a mouse in group B (400×). (PDF 674 kb) [file 12866_2019_1486_MOESM2_ESM.pdf]

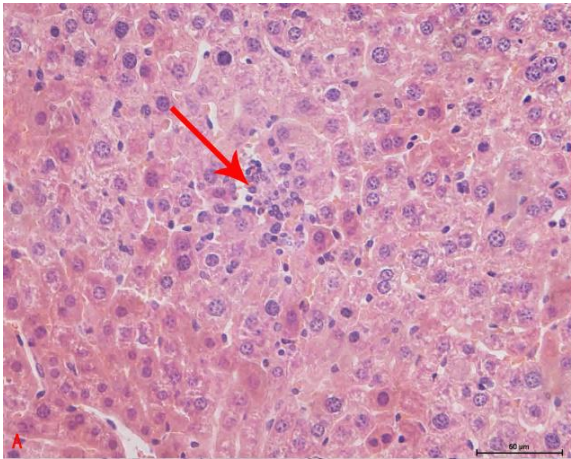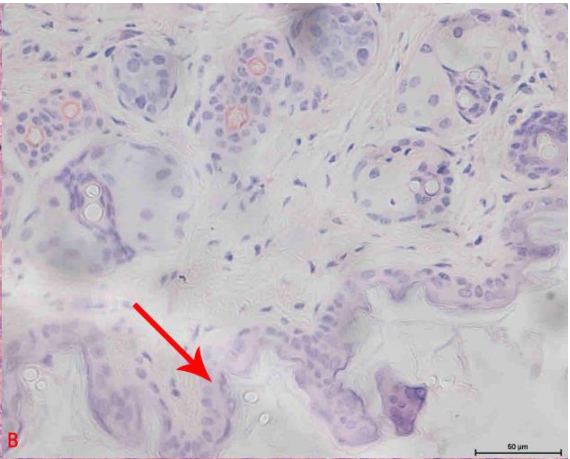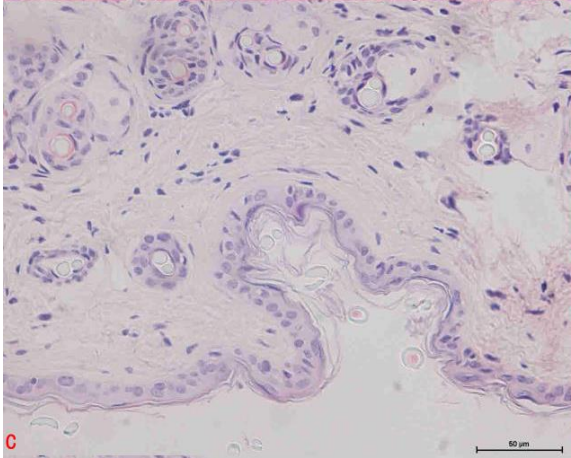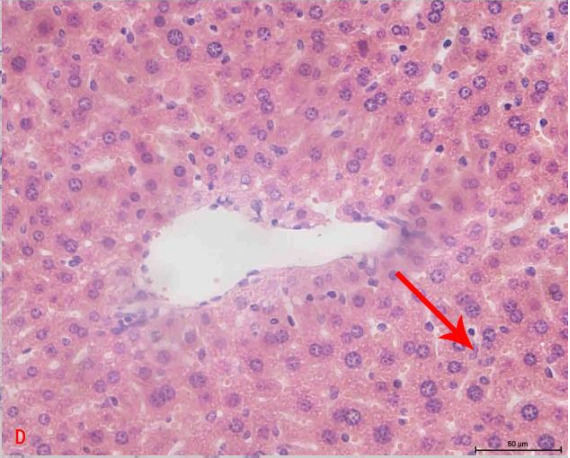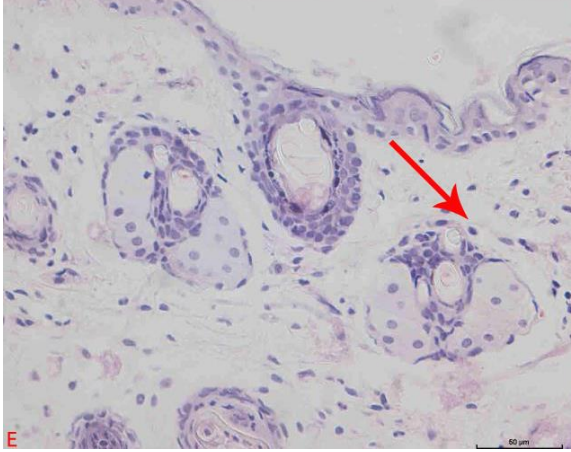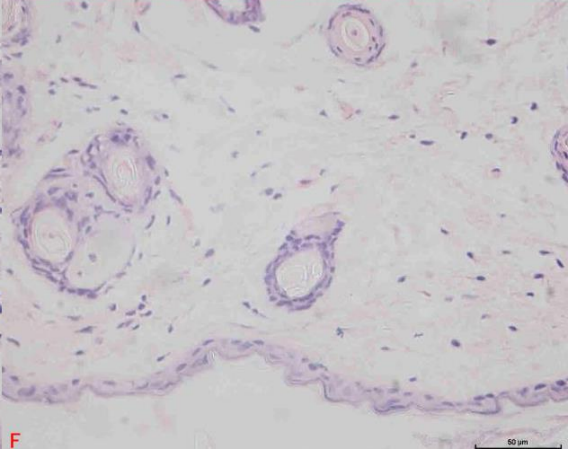

Supplement: Supplementary file 3 — Figure S3. Pathological sections of tissue damaged by Trichosporon domesticum (JYZ983) infection. A: Local necrosis and swelling of hepatocytes and infiltration of a small number of lymphocytes (400×); B: Thickening of the cuticle of the skin (400×); C: Normal structure (400×); D: Mild interstitial widening and swelling of hepatocytes (400×); E: Small amount of lymphocyte proliferation (400×); F: Normal structure (400×). (PDF 451 kb) [file 12866_2019_1486_MOESM3_ESM.pdf]

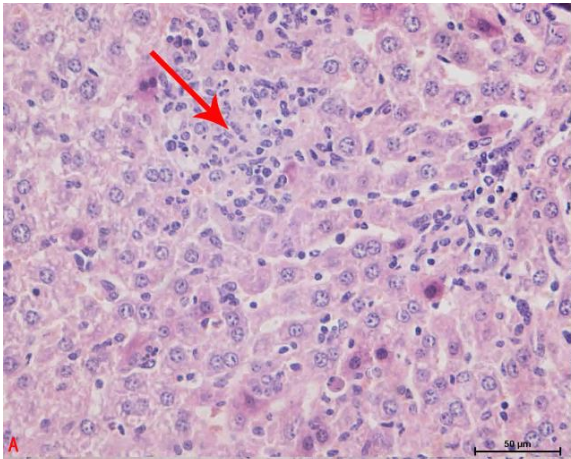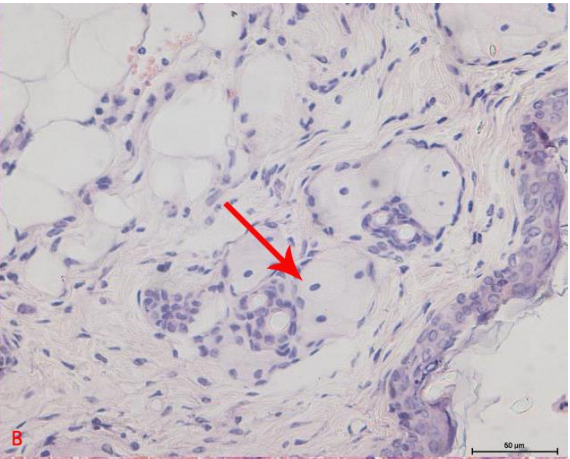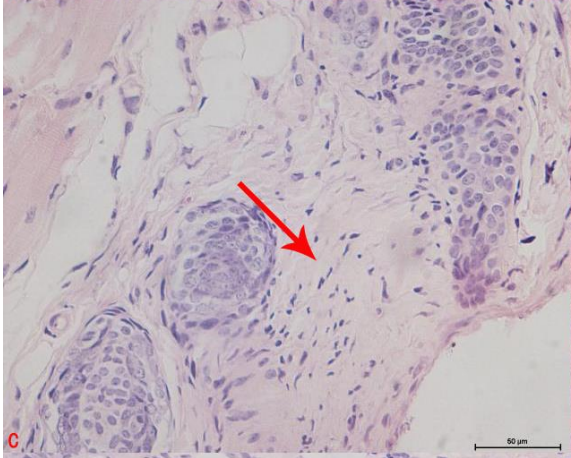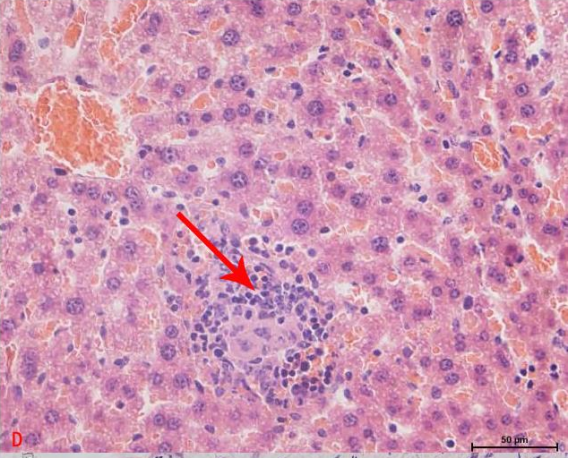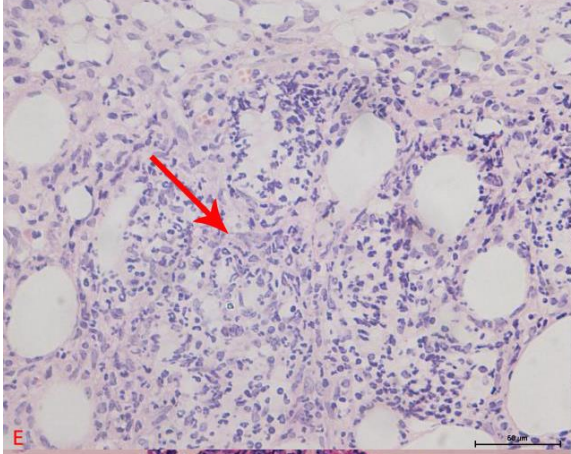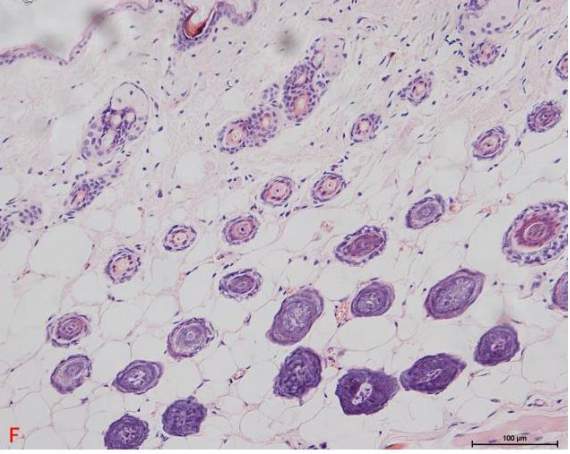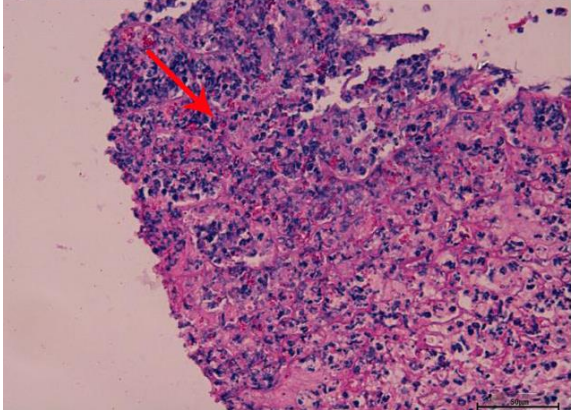

Supplement: Supplementary file 4 — Figure S4. Pathological sections of tissue damaged by Trichosporon guehoae (JYZ1221) infection. A: Hepatocyte necrosis, lymphocyte infiltration, hepatocyte swelling, and unclear hepatic cord structure (400×); B: Thickening of the cuticle of the skin and infiltration of reticular lymphocytes (400×); C: Normal structure (400×); D: Local necrosis of hepatocytes and diffuse congestion (400×); E: Necrosis of skin cells; F: Normal structure (400×); G: Spore stained with PAS in a lesion of the dermis of a mouse in group B (400×). (PDF 593 kb) [file 12866_2019_1486_MOESM4_ESM.pdf]

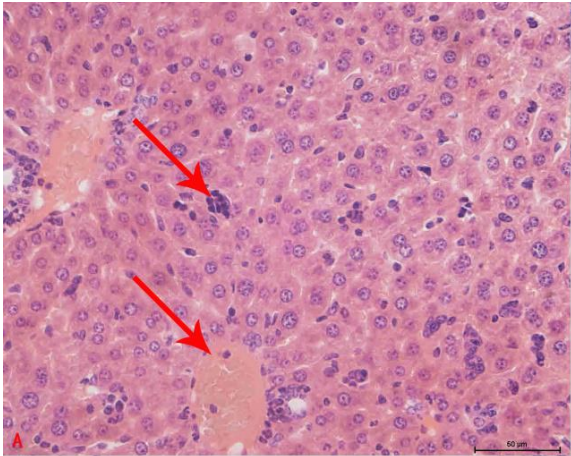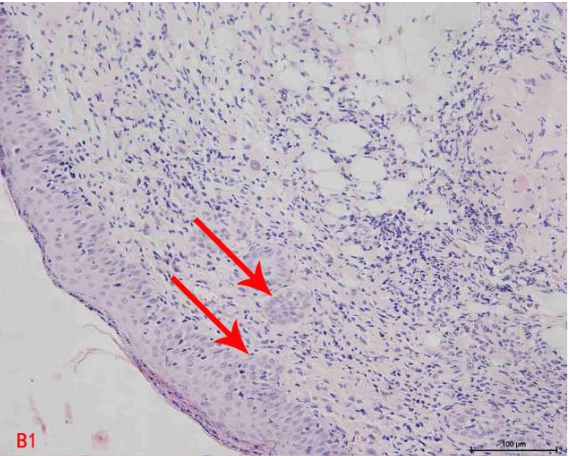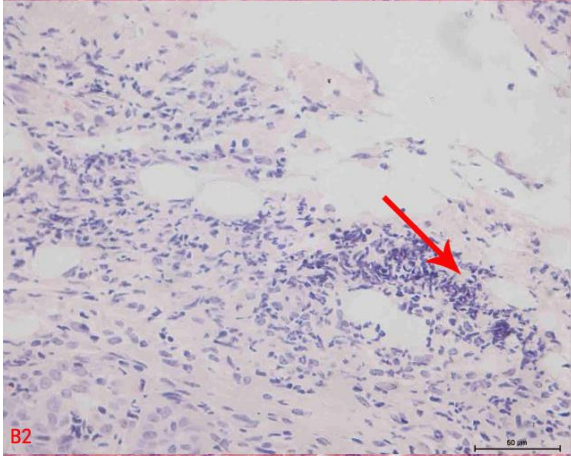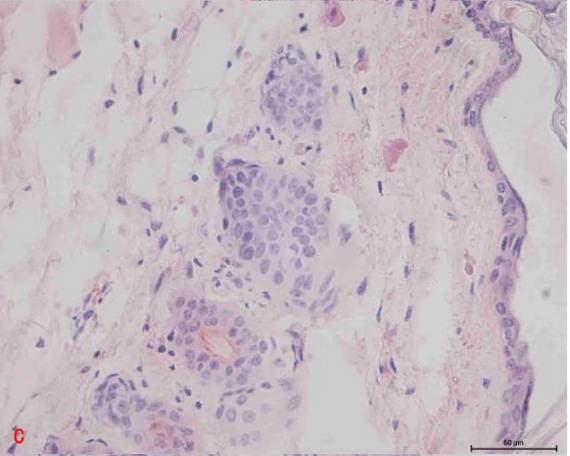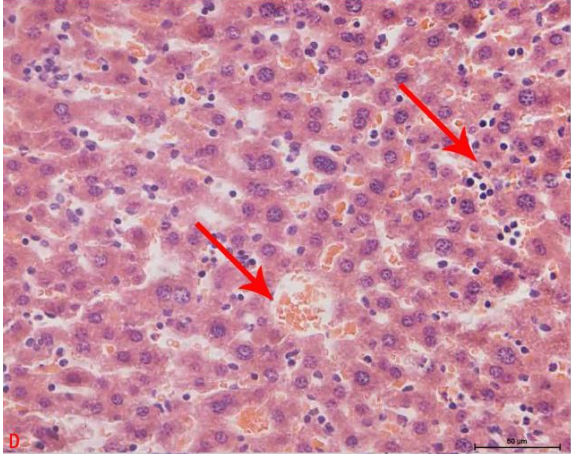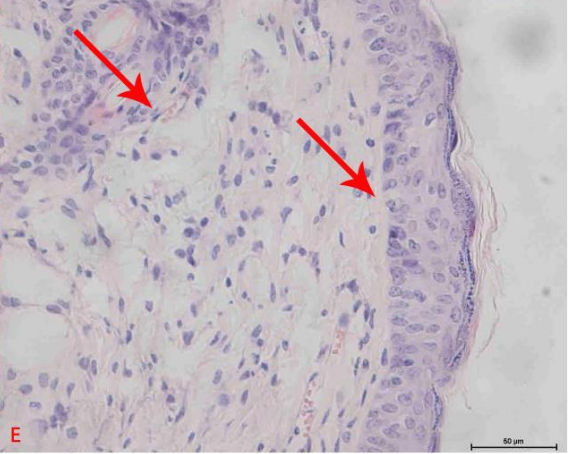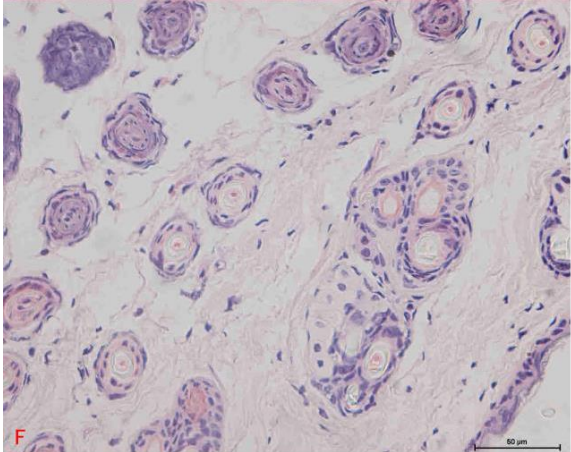

Supplement: Supplementary file 5 — Figure S5. Pathological sections of tissue damaged by Trichosporon jirovecii (JYZA10) infection. A: Central venous congestion of the liver, mild lymphocyte infiltration, and hepatocyte swelling (400×); B1: Thickening of the cuticle and necrosis of skin cells (200×); B2: Necrosis of reticular cell (400×); C: Normal skin structure (400×); D: Diffuse congestion of the liver and interstitial widening (400×); E: Thickening of the cuticle, infiltration of inflammatory cells, and local congestion of the reticular layer (400×); F: Normal skin structure (400×). (PDF 563 kb) [file 12866_2019_1486_MOESM5_ESM.pdf]

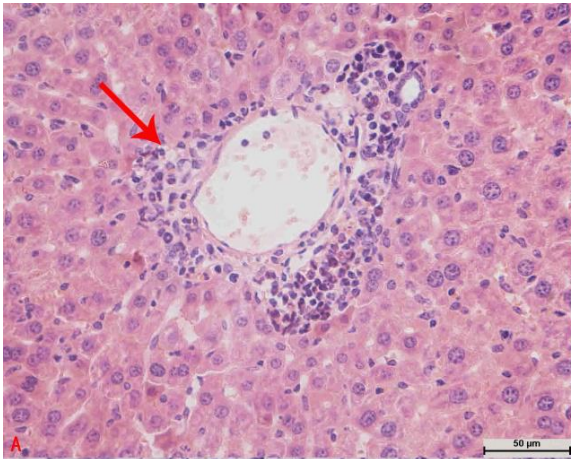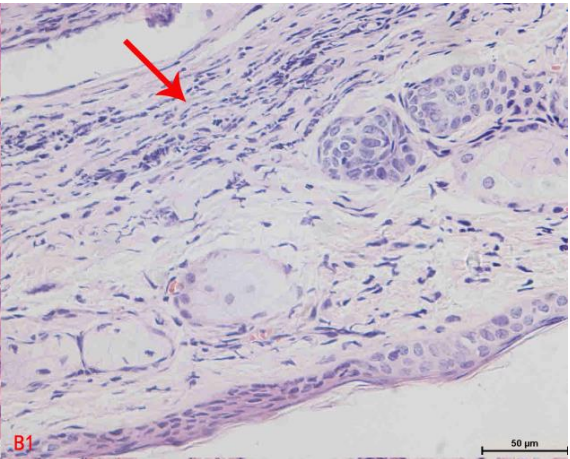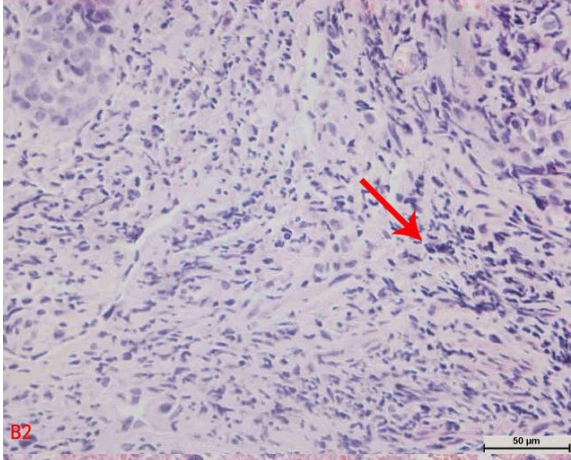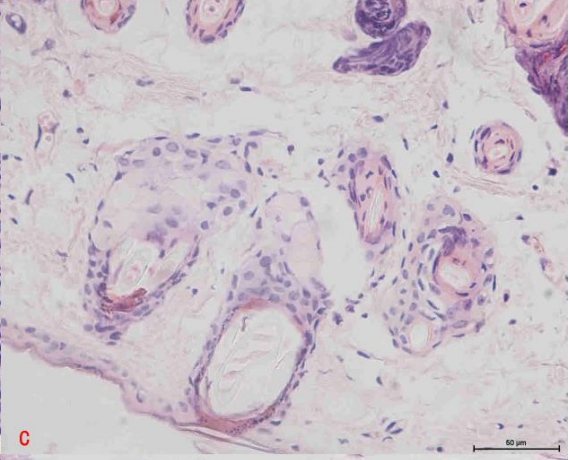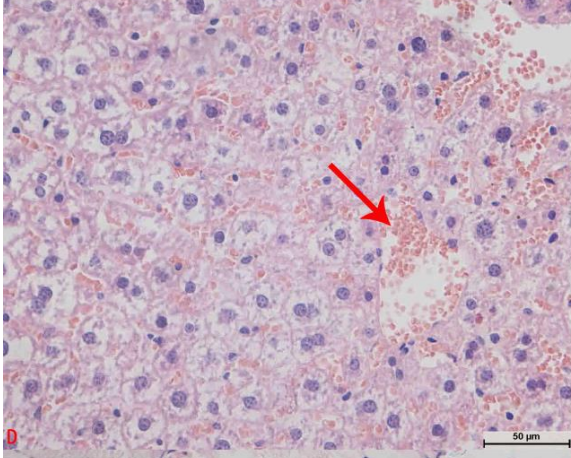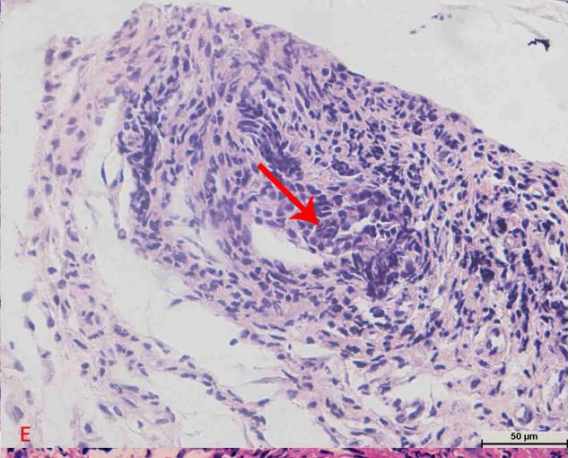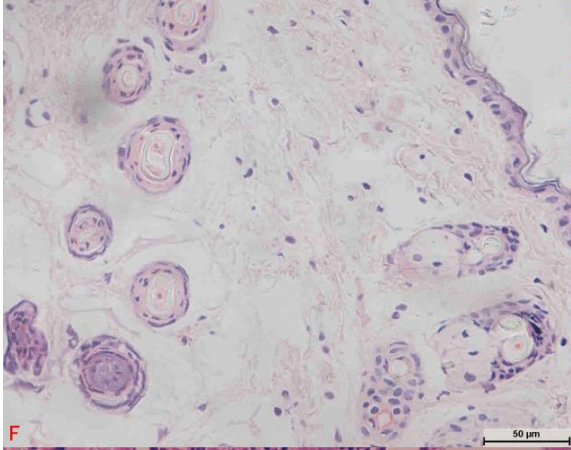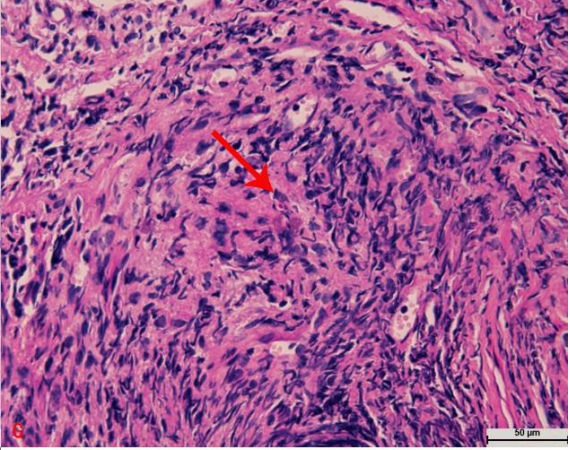

Supplement: Supplementary file 6 — Figure S6. Pathological sections of tissue damaged by Trichosporon cutaneum (JYZ030202) infection. A: Central venous congestion of the liver, necrosis of liver cells around the veins, and infiltration of lymphocytes (400×); B1: Necrosis of cells in the papillary layer and infiltration of lymphocytes (400×); B2: Necrosis of reticular cells in the skin and infiltration of lymphocytes (400×); C: Normal skin structure (400×); D: Central venous congestion of the liver, hepatic sinusoidal congestion, swelling of liver cells, and disorder of the hepatic cord (400×); E: Necrosis of reticular cells in the skin and proliferation of lymphocytes (400×); F: Normal skin structure (400×); G: Spore stained with PAS in a lesion in the dermis of a mouse in group B (400×); H: Spore stained with PAS in a lesion in the dermis of a mouse in group E (400×). (PDF 721 kb) [file 12866_2019_1486_MOESM6_ESM.pdf]

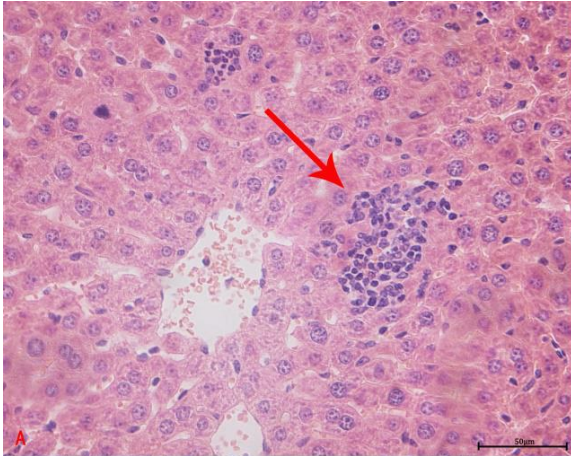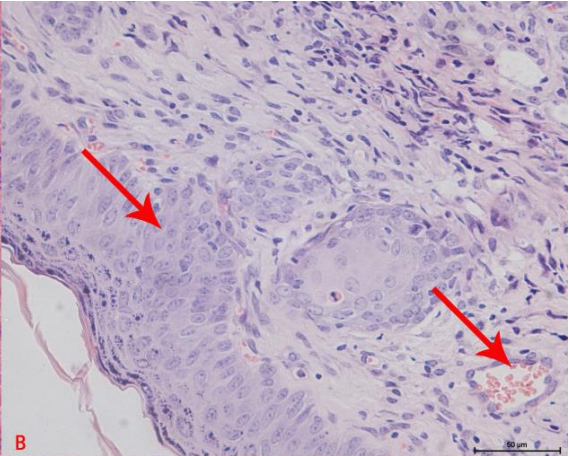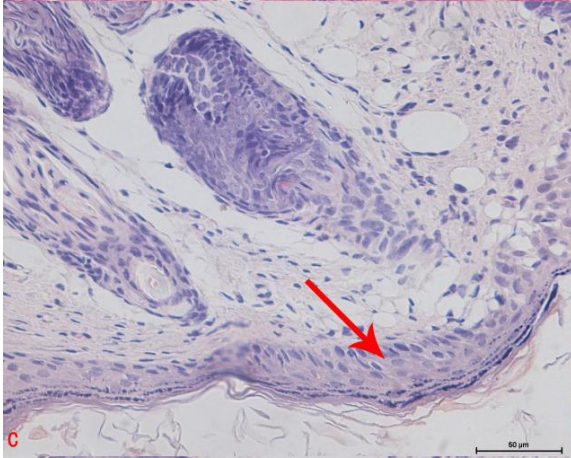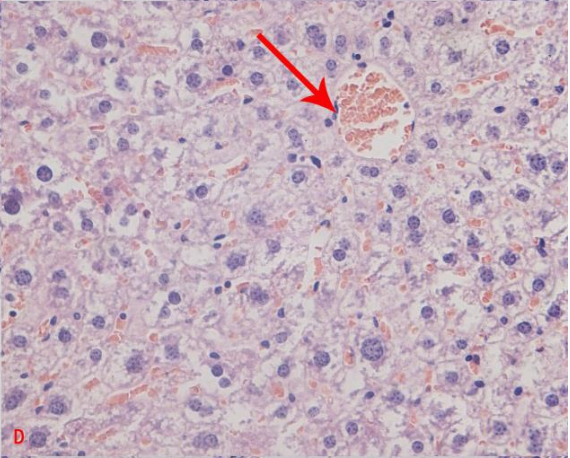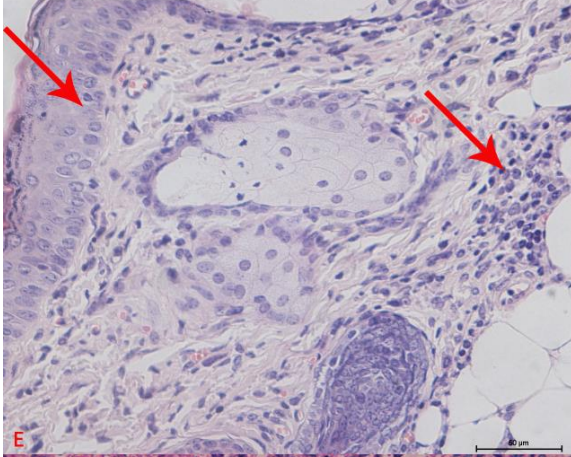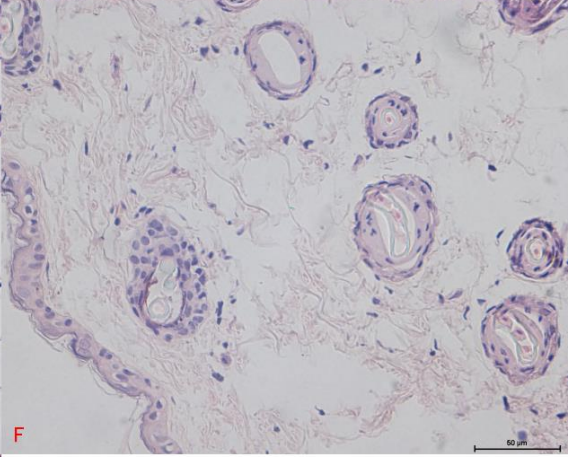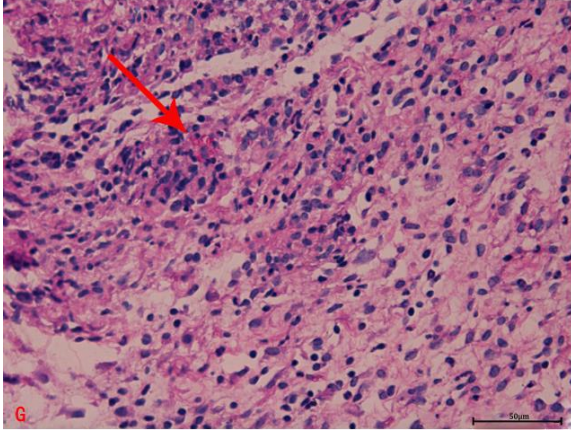

Supplement: Supplementary file 7 — Figure S7. Pathological sections of tissue damaged by Trichosporon shinodae (JYZ1223) infection. A: Central venous congestion of the liver, local necrosis of hepatocytes, and mild lymphocyte infiltration (400×); B: Thickening of the cuticle and granular layer, local necrosis of cells in the reticular layer, and proliferation of lymphocytes (400×); C: Normal skin structure (400×); D: Central venous congestion of the liver, hemorrhage of the hepatic sinusoids, swelling of liver cells, and infiltration of a small number of lymphocytes (400×); E: Slight thickening of the cuticle of the skin, local necrosis of reticular cells, and infiltration of lymphocytes (400×); F: Normal skin structure (400×); G: Spore stained with PAS in the dermis of a mouse in group B (400×). (PDF 610 kb) [file 12866_2019_1486_MOESM7_ESM.pdf]

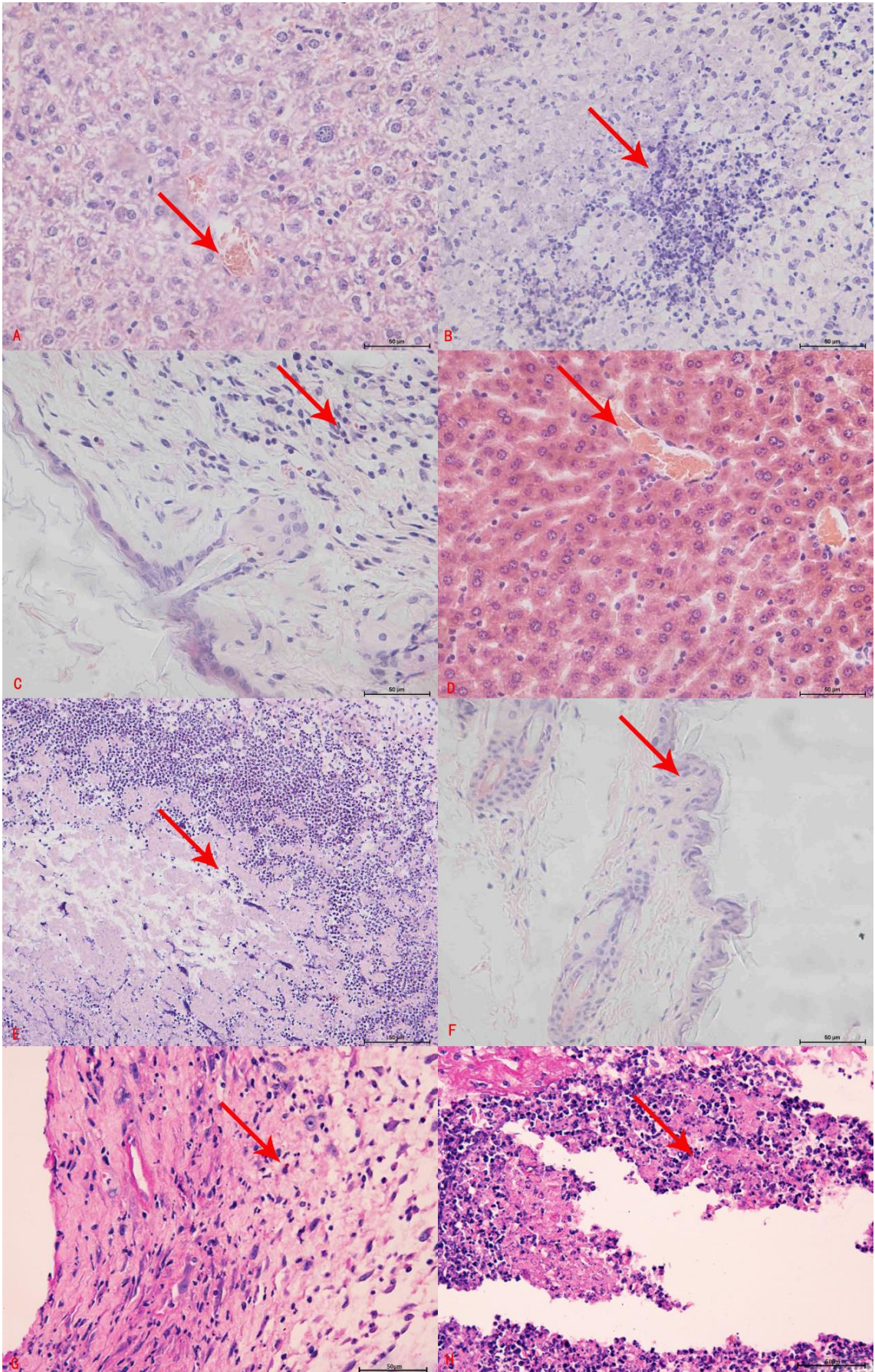

Supplement: Supplementary file 8 — Figure S8. Pathological sections of tissue damaged by Trichosporon middelhovenii (JYZ12922) infection. A: Diffuse congestion, venous congestion, hepatocyte swelling, unclear structure of the hepatic cord, and proliferation of lymphocytes (400×); B: Coagulative necrosis of reticular cells in the skin and proliferation of lymphocytes (400×); C: Thickening of the cuticle of the skin and proliferation of reticular lymphocytes (400×); D: Central venous congestion and interstitial widening (400×); E: Coagulative necrosis of skin cells, unclear structure of skin tissue, and proliferation of lymphocytes (400×); F: Thickening of the cuticle of the skin (400×); G: Spore stained with PAS in the dermis of a mouse in group C (400×); H: Spore stained with PAS in the dermis of a mouse in group B (400×). (PDF 679 kb) [file 12866_2019_1486_MOESM8_ESM.pdf]

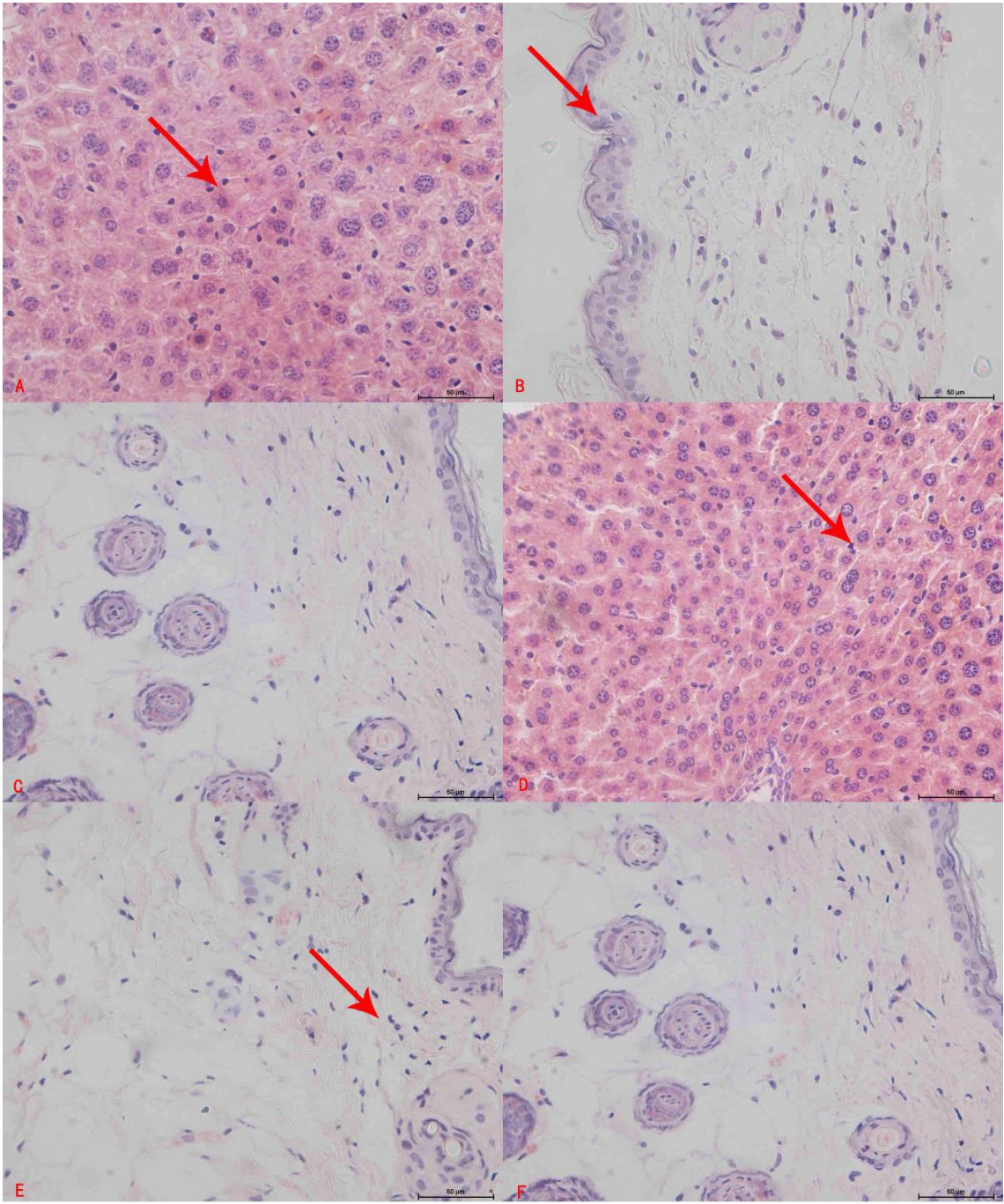

Supplement: Supplementary file 9 — Figure S9. Pathological sections of tissue damaged by Trichosporon moniliiforme (JYZ932) infection. A: Hepatocyte necrosis (400×); B: Thickening of the cuticle (400×); C: Basically normal structure of skin(400×); D: Proliferation of hepatocytes in the liver (400×); E: Proliferation of lymphocytes in the skin (400×); F: Basically normal structure of skin (400×). (PDF 430 kb) [file 12866_2019_1486_MOESM9_ESM.pdf]

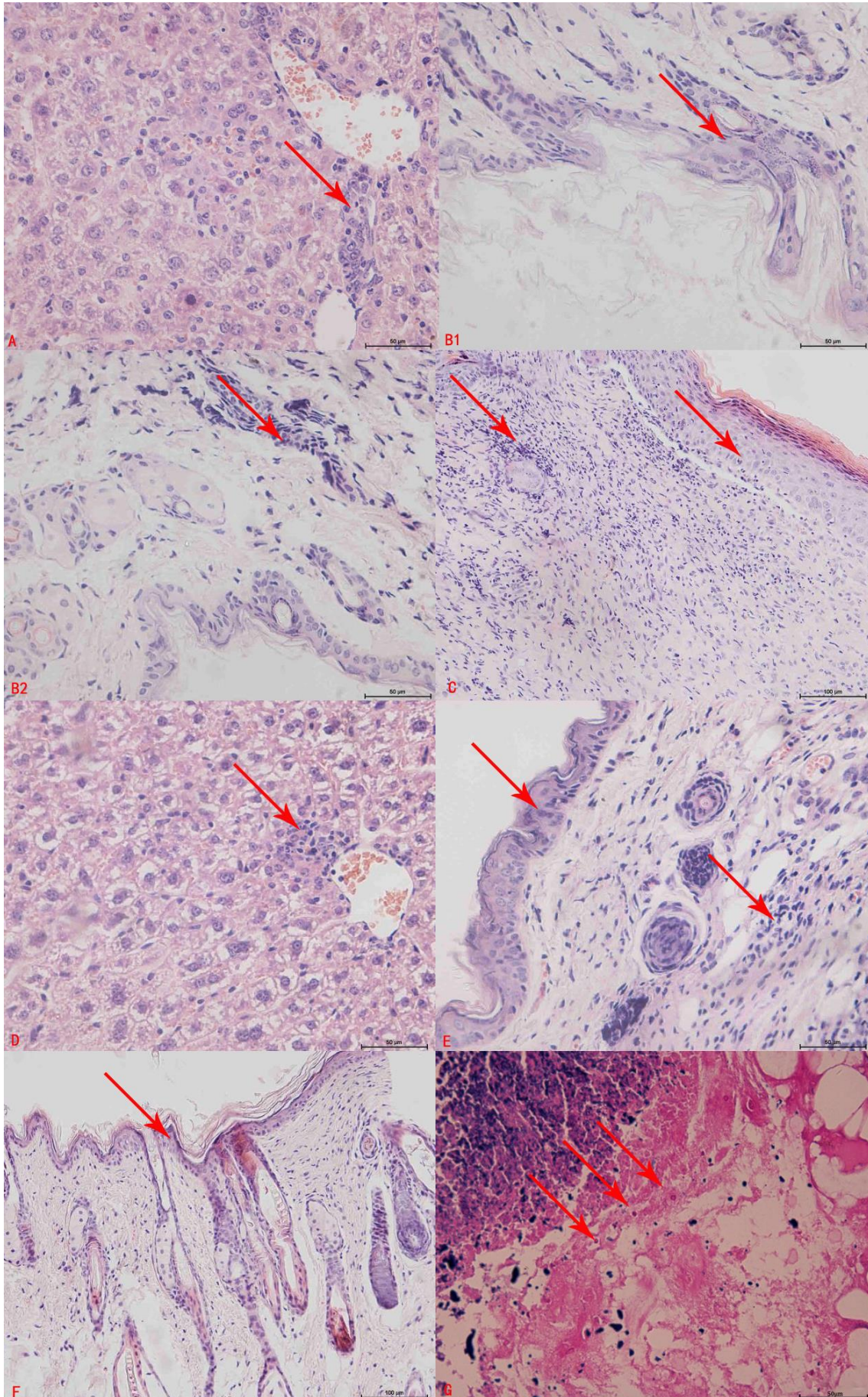

Supplement: Supplementary file 10 — Figure S10. Pathological sections of tissue damaged by Trichosporon laibachii (JYZ3252) infection. A: Central venous congestion of the liver, necrosis and swelling of hepatocytes, and unclear hepatic cord structure (400×); B1: Thickening of the cuticle of the skin (400×); B2: Mild necrosis of cells in the reticular layer (400×); C: Necrosis of skin cells, punctate infiltration of lymphocytes, and thickening of the granular layer (200×); D: Central venous congestion of the liver, hepatocyte necrosis, local infiltration of inflammatory cells, hepatocyte swelling, and unclear hepatic cord structure (400×); E: Thickening of the skin and local congestion (400×); F: Thickening of the cuticle (200×); G: Spore stained with PAS in a lesion in the dermis of a mouse in group C. (PDF 648 kb) [file 12866_2019_1486_MOESM10_ESM.pdf]
